# Sirtuins in intervertebral disc degeneration: current understanding

**DOI:** 10.1186/s10020-024-00811-0

**Published:** 2024-03-29

**Authors:** Jianlin Shen, Yujian Lan, Ziyu Ji, Huan Liu

**Affiliations:** 1 Department of Orthopaedics, Affiliated Hospital of Putian University, Putian, 351100 Fujian China; 2 Central Laboratory, Affiliated Hospital of Putian University, Putian, 351100 Fujian China; 3 School of Integrated Traditional Chinese and Western Medicine, Southwest Medical University, Luzhou, 646000 Sichuan China; 4 Department of Orthopaedics, The Affiliated Traditional Chinese Medicine Hospital, Southwest Medical University, Luzhou, 646000 Sichuan China; 5 The Third People’s Hospital of Longmatan District, Luzhou, 646000 Sichuan China

**Keywords:** SIRTs, Intervertebral disc degeneration, Autophagy, Apoptosis, Oxidative stress

## Abstract

**Background:**

Intervertebral disc degeneration (IVDD) is one of the etiologic factors of degenerative spinal diseases, which can lead to a variety of pathological spinal conditions such as disc herniation, spinal stenosis, and scoliosis. IVDD is a leading cause of lower back pain, the prevalence of which increases with age. Recently, Sirtuins/SIRTs and their related activators have received attention for their activity in the treatment of IVDD. In this paper, a comprehensive systematic review of the literature on the role of SIRTs and their activators on IVDD in recent years is presented. The molecular pathways involved in the regulation of IVDD by SIRTs are summarized, and the effects of SIRTs on senescence, inflammatory responses, oxidative stress, and mitochondrial dysfunction in myeloid cells are discussed with a view to suggesting possible solutions for the current treatment of IVDD.

**Purpose:**

This paper focuses on the molecular mechanisms by which SIRTs and their activators act on IVDD**.**

**Methods:**

A literature search was conducted in Pubmed and Web of Science databases over a 13-year period from 2011 to 2024 for the terms “SIRT”, “Sirtuin”, “IVDD”, “IDD”, “IVD”, “NP”, “Intervertebral disc degeneration”, “Intervertebral disc” and “Nucleus pulposus”.

**Results:**

According to the results, SIRTs and a large number of activators showed positive effects against IVDD.SIRTs modulate autophagy, myeloid apoptosis, oxidative stress and extracellular matrix degradation. In addition, they attenuate inflammatory factor-induced disc damage and maintain homeostasis during disc degeneration. Several clinical studies have reported the protective effects of some SIRTs activators (e.g., resveratrol, melatonin, honokiol, and 1,4-dihydropyridine) against IVDD.

**Conclusion:**

The fact that SIRTs and their activators play a hundred different roles in IVDD helps to better understand their potential to develop further treatments for IVDD.

**Novelty:**

This review summarizes current information on the mechanisms of action of SIRTs in IVDD and the challenges and limitations of translating their basic research into therapy.

## Introduction

Lower back pain (LBP) is a common pathologic condition of the musculoskeletal system. Approximately 80% of the global population experience acute or chronic LBP (Khan et al. 2017). The findings of several epidemiologic studies indicated that LBP is one of the leading causes of disability worldwide. This pain adversely affects the quality of life of patients and is a major economic burden for families and society worldwide (Zhang et al. 2020; Urits et al. 2019). Intervertebral disc degeneration (IVDD), which leads to disc herniation, spinal instability, vertebral slippage, and osteophytes, is considered the most important etiologic factor for LBP (Navone et al. 2017). However, the specific pathologic mechanisms of IVDD have not been elucidated, and the therapeutic effects of conservative treatment and surgery are not satisfactory (Vedicherla and Buckley 2017). Therefore, new treatment methods for IVDD are urgently required.

Silent information regulator 2 (Sir2)—a class of nicotinamide adenine dinucleotide (NAD+)-dependent deacetylase—was first identified in *Saccharomyces cerevisiae* (Michishita et al. 2005). Seven mammalian Sir2 homologous proteins (SIRT1–7), called Sir2-related enzymes (sirtuins), have been identified to date (Priyanka et al. 2016). The sirtuin family is characterized by highly conserved NAD+ -binding and catalytic structural domains and contains N- and C-terminals outside the catalytic core. Sirtuins are involved in the pathogenesis of various aging-related diseases by regulating inflammation, oxidative stress, and mitochondrial function (Priyanka et al. 2016; Yang et al. 2022). It is because of these powerful functions of SIRTs that they have now become a research hotspot for aging-related diseases. In recent years, a large number of studies on the role of SIRTs in IVDD have been reported, such as SIRTs can regulate autophagy, myeloid apoptosis, and extracellular matrix degradation, in addition to SIRTs can exert anti-inflammatory and anti-oxidative stress to alleviate IVDD, it is evident that the role of SIRTs in the pathogenesis of IVDD has become an active area of research. However, there is no literature that systematically summarizes the possible roles of the family of SIRTs in IVDD and organizes an effective pathway for the treatment of IVDD through the regulation of SIRTs. Therefore, the main objective of this paper is to review the current research on the mechanisms of SIRTs modulating disc degeneration and, based on this, to summarize the relevant aspects of treating IVDD by modulating SIRTs, including the challenges and limitations of translating basic research on SIRTs into therapy, which will provide new ideas for future research and clinical research areas to further contribute to the development of therapeutic potential for treating IVDD.

## Sirtuins: cellular regulators with multifaceted functions

### Overview of the sirtuin protein family and its role in cellular processes

Sir2 was first discovered during transcriptional silencing in *Saccharomyces cerevisiae* cells in the 1970s. It belongs to a highly conserved family of proteins that affect genome stability (Frydzinska et al. 2019), and their over-activation prolongs the lifespan in yeast (Kane and Sinclair 2018). The proteins homologous to Sir2 in eukaryotes were later called Sirtuins, which are class III histone deacetylases with NAD+ -dependent deacetylase activity. Sirtuins have a wide range of enzymatic activities as deacetylases, ADP ribosyltransferases, deglycosylases, and desuccinylases. Notably, binding to NAD+ is essential to all these functions (Vargas-Ortiz et al. 2019). Members of the sirtuin family regulate diverse cellular functions by acting on the post-translational modification of various proteins in organelles (Kanwal et al. 2019).

### Role of sirtuins in cellular senescence, metabolism, and stress response

SIRT1–7 are the seven isoforms in the sirtuin protein family, widely distributed in cells. SIRT1 and SIRT2 are localized in the cytoplasm. SIRT1, SIRT6, and SIRT7 are distributed in the nucleus, whereas SIRT3, SIRT4, and SIRT5 are found in the mitochondria (Taneja et al. 2021). SIRT1 is most closely related to Sir2 and has been extensively investigated. It is involved in numerous biological processes, such as the regulation of gene transcription, apoptosis, survival, inflammation, oxidative stress, cellular senescence, and tumor formation (Kuno et al. 2023). SIRT1 can be directly or indirectly involved in the regulation of adenosine 5′-monophosphate-activated protein kinase (AMPK) signaling pathway. SIRT1 can deacetylate not only histone proteins but also various non-histone proteins and transcription factors to influence multiple cellular processes, including DNA repair, stress response, and cell survival, to delay aging. SIRT1 is often referred to as the "longevity protein" because of its association with lifespan extension (Chen et al. 2020).

SIRT2 migrates to the nucleus during the G2/M phase, where it is involved in cell cycle regulation and intracellular transport (Lin et al. 2023) (Table 1). The activation of SIRT2 decreases reactive oxygen species (ROS) concentrations, deacetylates hepatic kinase B1, and promotes the activation of the AMPK pathway (Wu et al. 2021) involved in the regulation of cellular oxidative stress, autophagy, and apoptosis (Wang et al. 2019a). SIRT3 maintains mitochondrial energetic homeostasis by inducing the deacetylation and nuclear translocation of forkhead box O3a (FOXO3a) transcription factor to reduce ROS concentrations in cells (Tao et al. 2023). SIRT3 is also involved in tumorigenesis. SIRT4 plays a role in regulating energy metabolism and insulin secretion, and SIRT4 inhibits fatty acid oxidation in muscle and liver cells (Min et al. 2018).

**Table 1:** Sirtuins: cellular regulators with multifaceted functions

Authors (reference)	Type of study	Study design	Aim	Results	Conclusion
Kanwal et al. (2019)	An experimental study	Experimental in vivo study	To determine the role of Sirt6 and mitochondrial Sirt (Sirt3)in protecting a diabetic heart from developing cardiomyopathy	In cardiomyocytes, Sirt6 downregulates Keap1 expression and inhibits the binding of Kelch-like ECH-associated protein 1 (Keap1) to Nrf2, thereby stabilizing Nrf2 and activating the transcription of Nrf2-dependent genes, including Sirt3	Sirt6 and Sirt3 regulate each other's activity, which may be critical for coordinating cellular metabolism and maintaining the health of an organism
Kuno et al. (2023)	An experimental study	Experimental in vivo and in vitro study	To investigate whether SIRT1 counteracts doxorubicin-induced cardiotoxicity by mediating the phosphorylation of Ser139 of histone H2AX, a key signal in the DNA damage response	1) endogenous SIRT1 protein in cardiomyocytes counteracts doxorubicin-induced cardiotoxicity.2) SIRT1 mediates the DDR and protects cells from apoptosis by regulating histone H2AX phosphorylation via its deacetylation	SIRT1 counteracts doxorubicin-induced cardiotoxicity by mediating H2AX phosphorylation through its deacetylation in cardiomyocytes
Lin et al. (2023)	An experimental study	Experimental in vivo and in vitro study	Discovery of a mechanism by which hepatocyte SIRT2 regulates hepatic bone crosstalk	In SIRT2-deficient hepatocytes, LRG1 levels in sEVs are upregulated, leading to increased transfer of LRG1 to bone-marrow-derived monocytes (BMDMs), and in turn, to inhibition of osteoclast differentiation via reduced nuclear translocation of NF-κB p65	Hepatocyte SIRT2 regulates pro-osteoclast signaling of NF-κB p65 in osteoblasts via the sEV-LRG1 pathway

SIRT5 is the least known member of the sirtuin family and is involved in the urea cycle, glycolysis, fatty acid oxidation, and other processes that regulate malonylation of cytosolic and mitochondrial proteins (Fabbrizi et al. 2023). It is also involved in the development and progression of various cancers (Lagunas-Rangel 2023). SIRT6, highly similar in function to SIRT1, is an intracellular energy sensor and a major regulator of intracellular homeostasis (Chang et al. 2020). SIRT6 regulates genome integrity by participating in DNA repair and telomere maintenance. It modulates intracellular homeostasis by modulating glucose and lipid metabolism and inflammatory responses. In addition, SIRT6 is involved in various biological processes, such as immune response and cancer cell differentiation (Chen et al. 2021). SIRT7 is involved in gene regulation, genome stability, aging, and tumorigenesis (Tang et al. 2021). Moreover, it has also been recognized as a regulator of metabolism and stress responses (Bi et al. 2020).

## Link between sirtuins and IVDD

### Current understanding of IVDD pathogenesis and risk factors

**Fig. 1:**
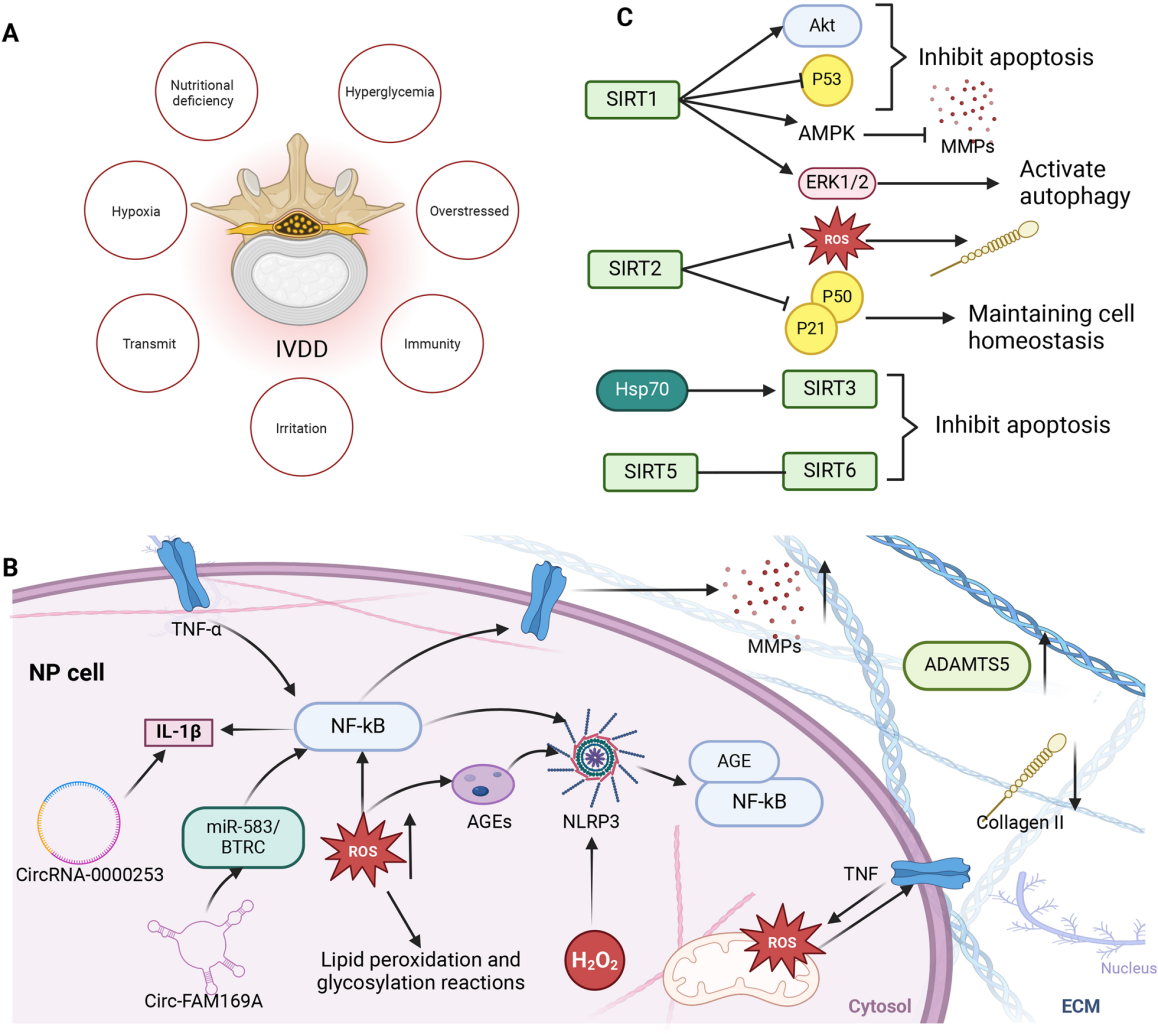
Correlation between SIRT and IVDD. **A** Risk factors for IVDD. Various factors such as nutritional deficiencies, hyperglycemia, stress, hypoxia, stress, genetics, and immunocompromise contribute to disc degeneration. **B** Pathogenesis of IVDD, which mainly involves NP cell metabolism (senescence and apoptosis), inflammation, ECM degradation, and oxidative stress. **C** Role of SIRT in NP cell survival and activity.SIRT mainly activates autophagy, maintains intracellular homeostasis, and inhibits apoptosis

The intervertebral disc consists of the nucleus pulposus (NP), annulus fibrosus, cartilaginous endplates, and capillary beds supplying nutrients. The disc is located between two adjacent vertebrae, where it serves as a weight-bearing device and maintains the stability and mobility of the vertebral body. The periphery of the NP is surrounded by a fibrous ring, and the extracellular matrix is rich in type II collagen and proteoglycans. The intervertebral disc is the largest avascular structure in the body and exchanges material through the surrounding capillary tissue. Therefore, it is highly susceptible to degenerative lesions (Kos et al. 2019). Various factors, such as nutritional deficiencies, hyperglycemia, excessive stress, hypoxia, stress, genetics, and low immunity, contribute to disc degeneration (Vergroesen et al. 2015) (Fig. 1A). This review mainly discusses the effects of related factors on the pathogenesis of IVDD from the metabolism (aging and apoptosis), inflammation, ECM degradation and oxidative stress of NP cells (Fig. 1B).

### Rationale for investigating sirtuins in IVDD

The function and number of NP cells or endplate chondrocytes are decreased in degenerated disc tissues. In addition, the production of extracellular matrix components, such as type II collagen and proteoglycans, is reduced, and their degradation is enhanced. This results in the secretion of matrix metalloproteinases (MMPs) by NP cells to maintain the homeostasis of the microenvironment (Dowdell et al. 2017). This process affects the development, metabolism, proliferation, and apoptosis of NP cells, which reduces the resistance of the disc to compression and leads to structural destruction. Various proteins of the sirtuin family promote intracellular anabolism, regulate apoptosis and autophagy, reduce the degradation of functional components of the extracellular matrix, and decrease the response of the intervertebral disc to stress and inflammatory factors, slowing down the process of disc degeneration (Guo et al. 1976).

### Presence and activity of sirtuin in intervertebral disc cells

The sirtuin family proteins ameliorate IVDD by promoting autophagy of NP cells and inhibiting apoptosis (Miyazaki et al. 2015) (Fig. 1C). Apoptosis and autophagy in NP cells are regulated by interrelated and interacting mechanisms. The sensitivity of NP cells to apoptosis increases when autophagic processes are blocked in NP cells (Guo et al. 1976). SIRT1 significantly inhibits apoptosis of NP cells through the Akt anti-apoptotic signaling pathway in the early stage of IVDD, slowing down IVDD (Wang et al. 2013).

Butein treatment of rats with hyperglycemia-induced disc degeneration induced an increase in Sirt1 and a decrease in acetylated p53. Therefore, butein may inhibit the acetylation of p53 by activating SIRT1 and protect NP cells from hyperglycemia-induced apoptosis and senescence (Zhang et al. 2019a). Quantitative real-time polymerase chain reaction (qRT-PCR)-based analysis revealed that resveratrol (3,5,4′ -trihydroxy-trans-stilbene) activates autophagy through the AMPK/SIRT1 signaling pathway, which attenuates TNF-α-induced MMP-3 expression in human cells (Wang et al. 2016a). SIRT1 increased the number of autophagosomes in NP cells by phosphorylating Erk1/2, thereby promoting autophagy and inhibiting apoptosis (Jiang et al. 2014). In addition, by evaluating the characteristics of the different disc degeneration stages, the early stage of disc degeneration was determined to be Pfirrmann grade III with normal disc height. The authors suggested that mildly degenerated NP cells may be a key target for molecular biological intervention therapy for disc degeneration. Notably, SIRT1 activates autophagy through the Akt/ERK signaling pathway and protects mildly degenerated human NP cells from apoptosis (He et al. 2021a).

SIRT2 overexpression significantly ameliorates ROS-induced IVDD and increases the concentrations of SOD1/2, type II collagen, and aggregated proteoglycans. In addition, SIRT2 overexpression in NP cells significantly downregulates the expression of p53 and p21. SIRT2 plays an important role in maintaining homeostasis by promoting anabolic metabolism and inhibiting catabolism in vivo (Yang et al. 2019). HSP70 can inhibit mitochondrial fission by upregulating SIRT3 expression, thereby attenuating compression-induced apoptosis in NP cells (Hu et al. 2022). SIRT5 is involved in maintaining mitochondrial homeostasis through its desuccinylase activity, and its expression was decreased in rat NP tissues under mechanical loading. Overexpression of Sirt5 effectively alleviated apoptosis and dysfunction of NP cells under mechanical stress, whereas its knockdown exacerbated apoptosis and dysfunction (Mao et al. 2023). Overexpression of SIRT6 inhibits apoptosis, replication of NP cells, and stress-induced premature senescence (Chen et al. 2018) (Table 2).

**Table 2:** Link between Sirtuins and IVDD

Authors (reference)	Type of study	Study design	Aim	Results	Conclusion
Guo et al. (1976)	An experimental study	Experimental in vivo and in vitro study	The aim of this report is to validate the expression of Sirt1 in different degrees of IVD degeneration	Sirt1 protein expression level decreased in the discs of high Pfirrmann grade and the score of histological morphology of human intervertebral disc is consistent with the Pfirrmann grade	Sirt1 is a protective mediator in IVD degeneration and the expression of Sirt1 decreases in degenerative disc. Activation of Sirt1 works on suppressing cellular senescence, promoting cell proliferation, and restraining the apoptosis of nucleus pulposus cells
Miyazaki et al. (2015)	An experimental study	In vitro study using human IVD nucleus pulposus (NP) cells	To explore the extent to which SIRT1 affects IVD cells	Recombinant human SIRT1 (rhSIRT1) inhibited the decrease in cell number and induced an increase in autophagic activity. rhSIRT1 decreased the incidence of apoptosis, with a decrease in anti-apoptotic Bcl-2 and an increase in pro-apoptotic Bax, cleaved caspase 3, and cleaved caspase 9 in NP cells under low-nutrient conditions, and these changes were affected by rhSIRT1 inhibition, suggesting that rhSIRT1-induced autophagy has an inhibitory effect on apoptosis	SIRT1 protects against nutrient deprivation-induced mitochondrial apoptosis through autophagy induction in human IVD NP cells
Wang et al. (2013)	An experimental study	in vitro study using human IVD nucleus pulposus (NP) cells	The present study was performed to determine whether degenerative human NP would express SIRT1, and to investigate the role of SIRT1 in NP cells apoptosis	SIRT1 mRNA and protein levels were higher in LVF disc NPs than in DDD disc NPs. the apoptosis rate of resveratrol-treated NP cells was much lower than that of SIRT1 siRNA-transfected or nicotinamide-treated NP cells. After transfection with SIRT1 siRNA, NP cells reduced Akt phosphorylation, whereas resveratrol phosphorylated Akt	SIRT1 plays a critical role in survival of degenerative human NP cells through the Akt anti-apoptotic signaling pathway
Zhang et al. (2019a)	An experimental study	Experimental in vivo and in vitro study	The aim of this study was to investigate the mechanism of action of diabetes mellitus on IDD	The acetylation of p53 was upregulated and the expression and activity of Sirt1 were downregulated in response to glucose in NP cells as well as in diabetic NP tissues. The upregulation of Sirt1 expression and activity reversed the acetylation of p53, which in turn suppressed apoptosis and senescence induced by high glucose in NP cells. Sirt1 plays a protective role against diabetic IDD in a rodent model	Hyperglycaemia may enhance apoptosis and senescence in NP cells both in vivo and in vitro via the Sirt1/acetyl-p53 axis. The activation of Sirt1 may reduce p53 acetylation and potentially protect NP cells against apoptosis and senescence
Wang et al. (2016a)	An experimental study	in vitro study using human IVD nucleus pulposus (NP) cells	The purpose of this study is to investigate whether RSV regulates TNF-α–induced matrix metalloproteinase-3 (MMP-3) expression	Resveratrol (RSV) induced autophagy in human NP cells, and inhibition of autophagy significantly abolished the inhibitory effect of RSV on TNF-α-mediated MMP-3 upregulation. In addition, RSV increased the expression of SIRT1, and knockdown of SIRT1 significantly inhibited RSV-induced autophagy in NP cells.RSV also activated AMP-activated protein kinase (AMPK), and inhibition of AMPK significantly suppressed RSV-induced SIRT1 expression	RSV attenuated TNF-α–induced MMP-3 expression in human NP cells by activating autophagy via AMPK/SIRT1 signaling pathway
Jiang et al. (2014)	An experimental study	in vitro study using human IVD nucleus pulposus (NP) cells	To explore whether autophagy is involved in the protective effect of SIRT1 against apoptosis in NP cells	Resveratrol could increase the protein expression of LC3-II/I and Beclin-1, and reduce apoptosis in degenerative NP cells. In contrast, the protein levels of LC3-II/I and Beclin-1 were down-regulated and apoptosis level was significantly up-regulated in treatment with nicotinamide or SIRT1-siRNA transfection	Autophagy may play an important role in IVD degeneration, and SIRT1 protected degenerative human NP cells against apoptosis via promoting autophagy
He et al. (2021a)	An experimental study	in vitro study using human IVD nucleus pulposus (NP) cells	This study tried to confirm the "early stage" of IVDD and the role of NP cell autophagy during IVDD as well as to determine the mechanism by which SIRT1 protects NP cells	Mildly degenerative (Pfirrmann grade III with normal height of intervertebral disc) NP cells may be the key target for biomolecular interventions in IVDD and that SIRT1 protects human mildly degenerative NP cells from apoptosis by activating autophagy via the ERK signalling pathway	SIRT1 inhibits apoptosis by promoting the autophagic flux in NP cells via the ERK signalling pathway during the key stage of degeneration
Yang et al. (2019)	An experimental study	in vitro study using nucleus pulposus (NP) cells	The aim of this study was to determine whether Sirt2 protected NP from degradation in IDD	The expression of Sirt2 markedly decreased in severe degenerated disc tissues. IL-1β significantly promoted the progress of IDD. Meanwhile, overexpression of Sirt2 could reverse the effects of IL-1β. Sirt2 overexpression obviously increased the production of antioxidant SOD1/2 and suppressed oxidative stress in the disc. Moreover, p53 and p21 could be significantly suppressed by Sirt2 overexpression	Sirt2 prevented NP degradation via restraining oxidative stress and cell senescence through inhibition of the p53/p21 pathway
Hu et al. (2022)	An experimental study	in vitro study using human and rat nucleus pulposus (NP) cells	The aim of this study was to investigate whether HSP70 could regulate the expression of SIRT3	Promoting HSP70 expression protects NP cells from abnormal mechanical loads in vitro and in vivo. HSP70 inhibits compression-induced mitochondrial fission by promoting SIRT3 expression, thereby attenuating mitochondrial dysfunction and reactive oxygen species production, and ultimately inhibiting the mitochondrial apoptotic pathway in NP cells	HSP70 inhibits mitochondrial fission by upregulating SIRT3 expression and attenuates NP cell apoptosis
Mao et al. (2023)	An experimental study	in vitro study using human and rat nucleus pulposus (NP) cells	The aim of this study was to investigate whether desuccinylation modifications of SIRT5 and proteins are involved in the regulation of mitochondrial compression-induced injury in NP cells	Reduced SIRT5 expression resulted in the increased succinylation of AIFM1, which in turn abolished the interaction between AIFM1 and CHCHD4 and thus led to the reduced electron transfer chain (ETC) complex subunits in NP cells. Reduced ETC complex subunits resulted in mitochondrial dysfunction and the subsequent occurrence of IDD under mechanical stress	Excessive mechanical loading increases succinylation levels in NP cells by decreasing the expression of the desuccinylase SIRT5, impairing mitochondrial function and contributing to subsequent IDD development
Chen et al. (2018)	An experimental study	Experimental in vivo and in vitro study	explored whether sirt6 influenced IDD	Sirt6 levels were reduced in senescent human NP cells. sirt6 overexpression prevented apoptosis as well as replication- and stress-induced premature senescence. sirt6 also activated autophagy in NP cells both in vivo and in vitro. the anti-aging and anti-apoptotic effects of sirt6 were partially reversed by 3-methyladenine (3-MA) and chloroquine (CQ)-mediated inhibition of autophagy and sirt6 regulated the expression of denaturation-associated proteins (DAPs), which were also shown to be associated with the development of the NP cells. SIRT6 regulates the expression of degeneration-associated proteins. In vivo, sirt6 overexpression attenuated IDD	sirt6 attenuates cellular senescence by triggering autophagy and reduces apoptosis, ultimately improving IDD

## Sirtuins and cellular senescence in IVDD

### Cellular senescence as a hallmark of aging and IVDD

Cellular senescence refers to an irreversible proliferative arrest of cells in the G0/G1 phase. It is morphologically characterized by cell enlargement, pigment accumulation in the cytoplasm, vacuole formation, decreased mitochondrial count, increased nuclear size, nuclear invagination, and chromatin condensation, ultimately leading to cell death (Sikora et al. 2014; Calcinotto et al. 2019). Another important feature of senescent cells is the secretion of various inflammatory factors, chemokines, growth factors, and tissue reconstruction proteases, which constitute the senescence-associated secretory phenotype. These factors are extensively involved in multiple biological processes, such as inflammatory response, cell proliferation, and cell migration (Calcinotto et al. 2019).

The local metabolic state changes when intervertebral NP cells undergo aging. This change is manifested as a decrease in anabolism and an increase in catabolism, resulting in pathologic changes such as increased extracellular matrix degradation and decreased NP water content (Stich et al. 2020). The number of SA-β-gal staining-positive cells significantly increases in the NP tissues of patients with IVDD, and their number correlates positively with the Pfirrmann grade on magnetic resonance imaging of the intervertebral disc and negatively with the number of Ki67-positive (proliferating) cells (Li et al. 2019a; Novais et al. 2019; Che et al. 2019). Therefore, NP cell senescence is positively correlated with the severity of IVDD.

### SIRT1-mediated regulation of senescence pathways

Aging involves the loss and degradation of various physiologic functions in the organism, and these changes are mainly reflected in the loss of cells and constitutive substances in the tissues and slowing down of the metabolic rate. Therefore, maintaining the normal function of mitochondria to ensure optimal metabolism is critical to slow down the aging process (You and Liang 2023).

Oxidative metabolism occurs through the peroxisome proliferator-activated receptor γ coactivator 1 (PGC1)-α-dependent and PGC1-α non-dependent pathways in mitochondria, and Sirt1 plays an important role in regulating these pathways. PGC1-α and cytoplasmic Sirt1 are localized in the mitochondrial matrix (Aquilano et al. 2013). Sirt1 activates PGC1-α by acetylation, and both of them further co-activate mitochondrial transcription factor A (TFAM). The activated TFAM binds to the D-loop region of mtDNA to form a complex, which regulates mitochondrial DNA replication and transcription, thereby affecting mitochondrial biosynthesis (Yuan et al. 2016). The key protein in the PGC1-α non-dependent pathway is hypoxia-inducible factor 1 (HIF1)-α, which blocks TFAM transcription. The protein also binds to and inhibits PGC1-β activity (Yu et al. 2018). This is another positive regulator of mitochondrial biosynthesis. However, reduced Sirt1 expression increases the stabilization of HIF1-α, which downregulates mitochondrial bioactivity (Bellafante et al. 2014).

**Fig. 2:**
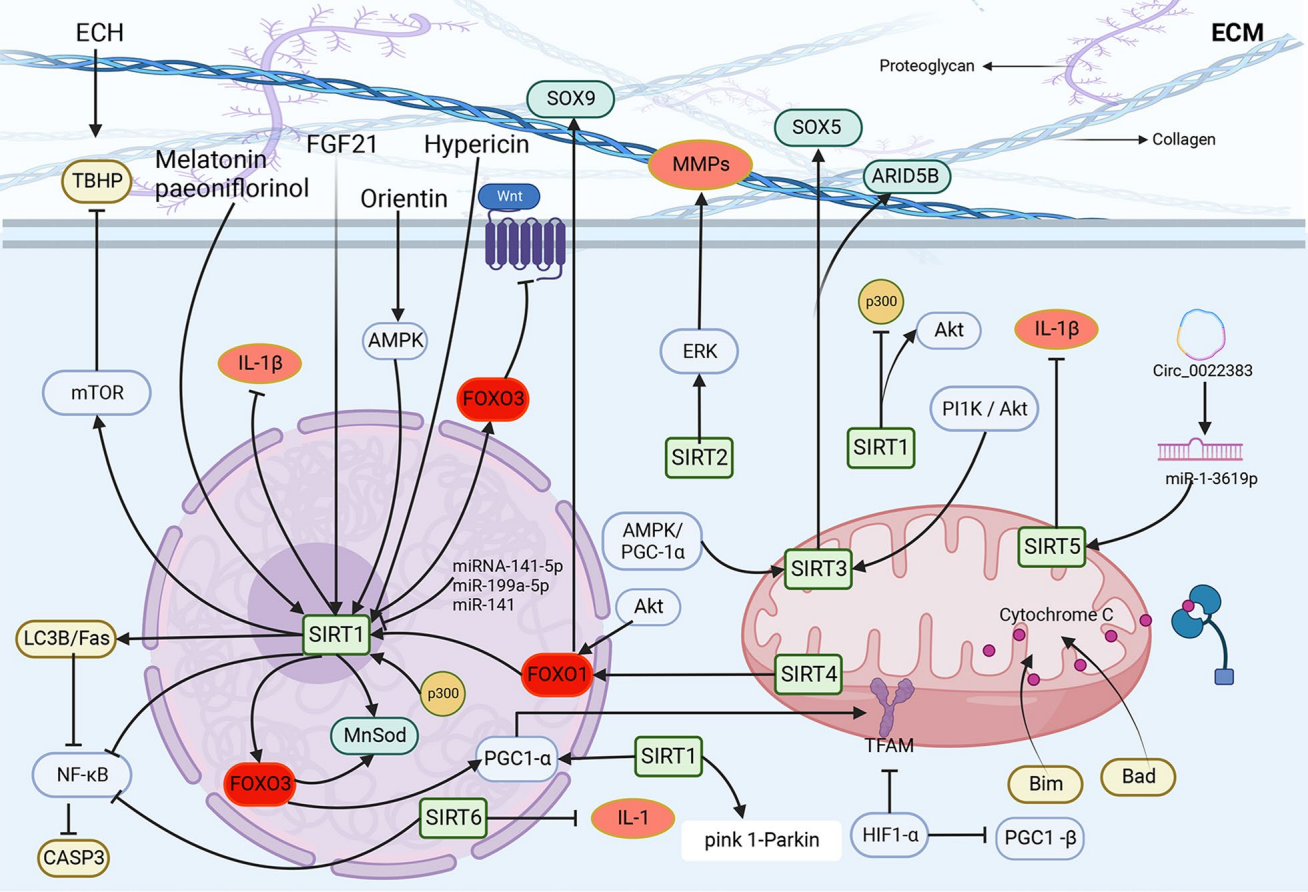
SIRT in IVDD cell metabolism.SIRT1 alleviates cellular senescence and apoptosis by regulating PGC-α,FOXO3, IL-β, Akt, mTOR AMPK, and NF-KB.SIRT2 regulates ERK.SIRT3 is activated by p11k/Akt and AMPK/PGC-1α and releases SOX5 and ARID5B.SIRT4 activates FOXO1 to maintain ECM homeostasis, thereby maintaining disc integrity.SIRT5 and SIRT6 inhibit the release of inflammatory factors.SIRT5 and SIRT6 inhibit the release of inflammatory factors.SIRT6 inhibits the release of inflammatory factors. SIRT4 activates FOXO1 to maintain ECM homeostasis, thereby maintaining disc integrity.SIRT5 and SIRT6 inhibit the release of inflammatory factors. Some miRNAs can also activate SIRT to regulate cellular metabolism

FOXO3 has multiple biological effects, including resistance to oxidative stress damage. The expression of Sirt1 and FOXO3 decreases in cardiac microvascular endothelial cells of aging mice (Lin et al. 2014). Sirt1 regulates ROS generation under oxidative stress by activating the expression of the manganese-containing superoxide dismutase (MnSOD; an antioxidant-acting superoxide dismutase in mammals) transcription factor through the deacetylation of FOXO3 (Emidio et al. 2014). Sirt1 deacetylates FOXO3 to restore and enhance its transcriptional activity and upregulates the expression of MnSOD to achieve antioxidant effects (Emidio et al. 2014). In addition, activated FOXO3 induces the transcription of PGC1-α and regulates mitochondrial function. Sirt1 acts as a deacetylase and has a delaying effect on aging. However, the regulatory pathway is affected by various factors, and the current studies on its role in aging-related pathways are in the speculative stage. (Fig. 2).

### Potential implications for delaying or reversing disc degeneration

High-intensity stress inhibits mitochondrial function and promotes ROS production. ROS damage intracellular DNA and other important biomolecules, promoting premature cell death (Benkafadar et al. 2019) and accelerating IVDD (Fearing et al. 2018). Consequently, the NF-κB pathway is activated, which exacerbates the senescence of NP cells. The Akt/FOXO1 pathway acts upstream of SIRT1 to modulate its expression and regulate H_2_O_2_-induced senescence in rat bone marrow cells (Wu et al. 2022). Deficiency of PTEN-induced kinase 1 (PINK1), a key mitotic regulator, impairs mitosis and diminishes the protective effect of SIRT1 against compression-induced senescence in myeloid cells. Wang et al. (2020a) reported that SIRT1 diminishes high-intensity compression-induced senescence in human NP cells through the PINK1/Parkin axis, a central mitotic mechanism. Furthermore, SIRT1 inhibits apoptosis by promoting autophagy (Jiang et al. 2014). SIRT1 overexpression attenuates decrease in autophagy and increase in NP cell senescence induced by high stress. In addition, SIRT1 overexpression significantly increases the LC3B/Fas complex formation, which rapidly inhibits the activation of the NF-κB pathway and thus CASP3 cleavage. LC3B silencing attenuates the inhibition of the NF-κB signaling pathway, partially promoting CASP3 cleavage and inhibiting NP cell senescence under high-intensity stress conditions (Zhuo et al. 2021). Overexpression of p300 promotes NP cell proliferation and autophagy. P300 enhances FOXO3 expression by binding to the SIRT1 promoter, leading to inactivation of the Wnt/β-cyclin pathway. Moreover, p300 disrupts the Wnt/β-linker pathway through the FOXO3/SIRT1 axis, thereby delaying the progression of IVDD (Hao et al. 2022).

Oxidative stress-induced myeloid cell senescence and apoptosis are mediated by the dysregulation of SIRT3 and the secondary imbalance of mitochondrial redox homeostasis. SIRT3 expression decreases with the progression of IVDD in humans. Wang et al. reported that in vitro silencing of SIRT3 expression reduces cellular resistance to oxidative stress and promotes senescence and apoptosis in rat myeloid cells. In contrast, activation of SIRT3 significantly inhibits oxidative stress-induced myeloid cell senescence and apoptosis and delays IVDD (Wang et al. 2018a). Lin et al. (2021a) reported a similar effect of SIRT3 on oxidative stress-induced myeloid cell senescence (Fig. 2).

## Anti-inflammatory effects of sirtuins in IVDD

### Role of inflammation in IVDD progression

Inflammation is a pathologic process initiated in response to infection or tissue damage. Inflammation is a key factor in the process of IVDD (Jin et al. 2019; Han et al. 2019). The concentrations of various pro-inflammatory cytokines, including interleukin (IL)-1α, IL-1β, IL-6, IL-17, and tumor necrosis factor (TNF)-α, significantly increase in the degenerated discs with the progression of IVDD (Cai et al. 2017; Mouser et al. 2019). These cytokines generate local autoimmune inflammatory reactions and enhance the catabolism of extracellular matrix in the intervertebral disc, leading to disc dysfunction and structural changes. IL-1β concentration increases in degenerated intervertebral discs and is directly proportional to the severity of IVDD. IL-1β directly inhibits extracellular matrix synthesis and forms a positive feedback loop. The loop stimulates the release of other inflammatory mediators and the synthesis of MMPs, increasing local catabolism in the intervertebral disc (Jin et al. 2019). IL-1β stimulation of human NP and annulus fibrosus cells substantially increases the secretion of IL-6, IL-8, and IL-17. Therefore, IL-1β may act as a key initiator of the inflammatory cascade by promoting the release of IL-6, IL-8, and IL-17 (Cai et al. 2017).

COX-2 plays an important role in inflammation. COX-2 is induced in many cell types in response to stimulation by inflammatory cytokines such as IL-1β and tumor necrosis factor alpha (TNF-α), as confirmed in IVDD (Miyamoto et al. 2000). COX-2 is the gene that produces PGE_2_ in cells, and when stimulated, activation of COX-2 leads to the production of PGE_2_ in IVDD and causes deleterious pathophysiologic effects such as inflammation20839316. PGE_2_ inhibits the synthesis of aggregated glycans in NPCs, leading to extracellular matrix disruption in IVDD (Lowe et al. 1996). It has been reported Propionibacterium acnes-induced activation of iNOS/NO and COX-2/PGE_2_ via the ROS-dependent NF-κB pathway may be responsible for the pathology of IVDD (Lin et al. 2018a). As IVDD progresses, the presence of COX-2 gradually increases (Lin et al. 2018a). Therefore, COX-2 is not only a cell signaling factor, but also a well-known pathogen for IVDD.

Chen et al. (2017) performed enzyme-linked immunosorbent assay (ELISA) and found that serum IL-21 concentration in patients with lumbar disc herniation was significantly higher than that in patients without disc herniation. Gorth et al. (2019) investigated the role of IL-1 in age-associated IVDD and found that IL-1α/β double knockout mice showed a significant reduction in blood concentrations of interferon-γ, IL-5, and IL-15. The number of SA-β-gal-positive cells significantly increased after TNF-α and IL-1β treatment of rat NP cells (Li et al. 2019b). Circ-FAM169A promotes IDD by regulating NF-κB pathway-induced IL-1β and TNF-α production via the miR-583/BTRC signaling pathway, upregulating the expression of MMP13 and ADAMTS5, and downregulating the expression of collagen II and aggrecan (Guo et al. 2020). In addition, some inflammatory factors, such as IL-1β, IL-6, and TNF-α, stimulate the sinusoidal nerve endings that grow into the intervertebral discs, which, in turn, trigger the clinical symptoms of radicular pain (Feng et al. 2016). Overall, inflammatory factors are powerful pro-cellular senescence factors, leading to IVDD and chronic lower back pain.

### Effect of sirtuins on the NF-κB signaling and inflammatory responses

Classical NF-κB is a P50–P50/ P60–P60 homodimer or a P50–P65 heterodimer formed by P50 and RelA/P65. The P50–P65 heterodimer plays a major physiologic role in vivo. NF-κB mostly binds to its inhibitory proteins in the cytosol to form inactive complexes (Capece et al. 2022). NF-κB inhibitory protein disassociates from the complex after cell stimulation and translocates into the nucleus to regulate the transcriptional activation of the target genes. However, NF-κB heterodimer must undergo some post-translational modification before exerting the regulatory effect (Maubach et al. 2022). Reversible acetylation/deacetylation is an important post-translational modification of NF-κB, which can regulate a variety of physiologic activities, including chromatin aggregation and gene transcription (Zhao et al. 2023). NF-κB transcription factors including P65, can precisely regulate NF-κB transcriptional activation through histone acetyltransferases and deacetylases (Guldenpfennig et al. 2023).

Yeung et al. (2004) evaluated the effect of SIRT1 on NF-κB signaling and inflammatory response and suggested that SIRT1 directly deacetylates the P65/RelA subunit of NF-κB and decreases the level of its acetylation, inhibiting the transcriptional function of downstream factors. SIRT1 directly acts on P65/RelA and reduces the acetylation level of Lys310, inhibits its transcriptional activity, and downregulates the expression of downstream genes. They used the luciferase reporter gene system and detected that the intracellular SIRT1 overexpression inhibited the transcriptional activity of NF-κB. These results were similar to those reported by Yeung et al. (2011). The knockdown of SIRT1 can lead to NF-κB hyperacetylation. Therefore, SIRT1 is a key enzyme that catalyzes the deacetylation of NF-κB (Yu and Auwerx 2010).

Overexpression or activation of SIRT1 can inhibit the inflammatory response, whereas its deletion can enhance the inflammatory response (Yoshizaki et al. 2010). Inflammatory stimuli can phosphorylate P300 through the mitogen-activated protein kinase (MAPK) signaling pathway and activate its histone acetyltransferase activity. The phosphorylated P300 catalyzes the acetylation of NF-κB, increases the binding of NF-κB to κB sequences, and initiates the NF-κB-mediated transcription of pro-inflammatory genes (Yang et al. 2022). SIRT1 catalyzes the deacetylation of NF-κB and restricts the overactivation of NF-κB, thereby reducing the inflammatory response. The results of transgenic animal experiments were consistent with those of cell culture experiments in this context. Lipopolysaccharide-induced NF-κB activation and the expression of various pro-inflammatory cytokines were significantly increased in SIRT1-excluded mouse RAW264.7 macrophages (Yoshizaki et al. 2009). Myeloid cell-specific SIRT1 knockout mice are highly sensitive to local or systemic endotoxin challenges (Schug et al. 2010). Reduced SIRT1 expression leads to inflammation and macrophage accumulation in the adipose tissue. The knockdown of SIRT1 in the adipose tissue stimulates the activation of NF-κB and highly acetylated H3K9 in histones, which, in turn, promote the activation of inflammatory genes (Gillum et al. 2011). The levels of SIRT1 protein in lung cells were significantly decreased in patients with COPD. This decrease was accompanied by increased levels of NF-κB acetylation and a corresponding increase in the concentrations of NF-κB-dependent pro-inflammatory cytokines (Rajendrasozhan et al. 2008).

**Fig. 3:**
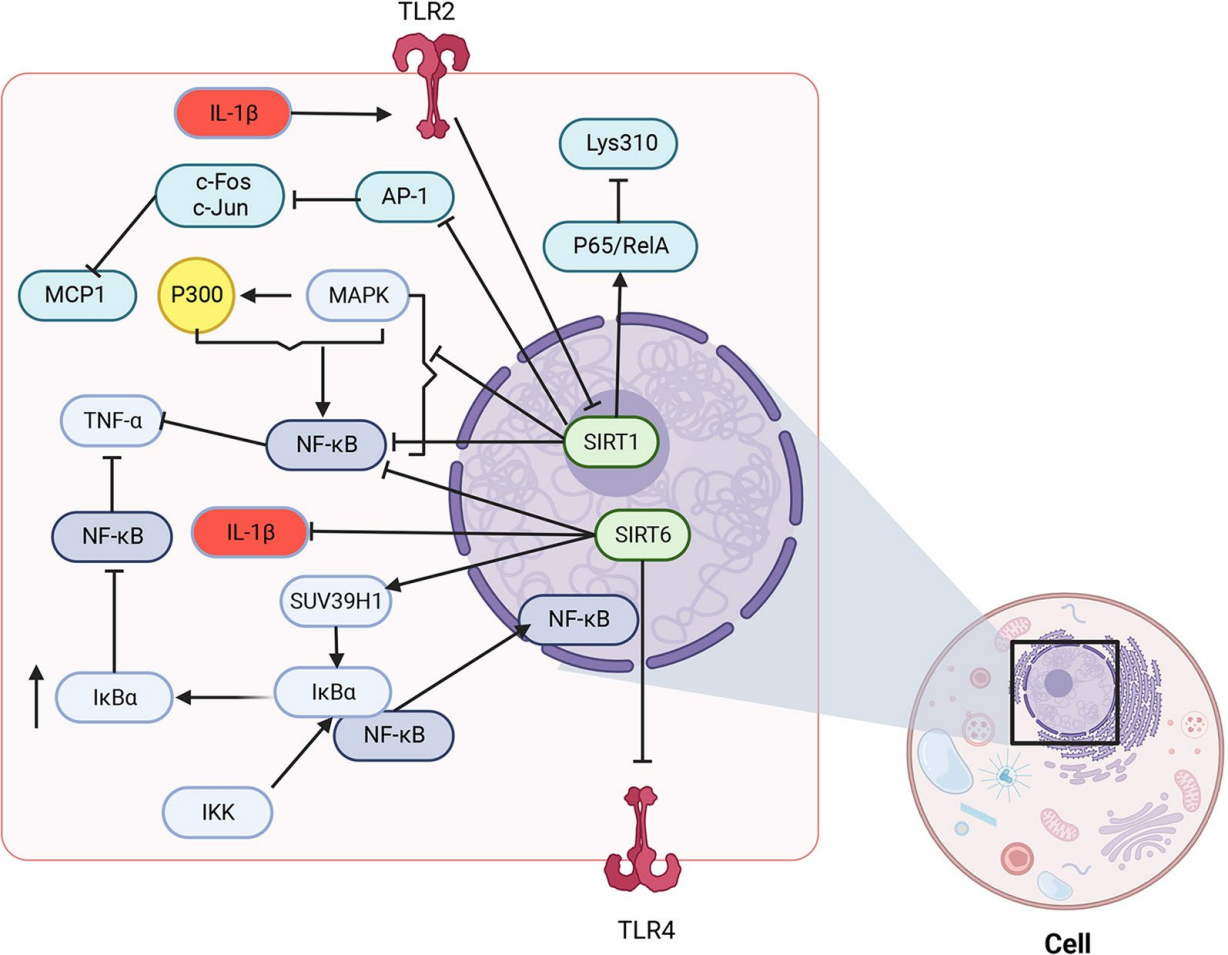
Anti-inflammatory effects of SIRT in IVDD.SIRT1 and SIRT6 mainly exert anti-inflammatory effects in IVDD.SIRT1 and SIRT6 can inhibit the NF-κB signaling pathway as well as the release of inflammatory factors through various pathways to exert anti-inflammatory effects

SIRT6 is also closely related to the NF-κB signaling pathway. Cells transfected with a vector expressing SIRT6 showed suppressed NF-κB transcriptional activity (Lim et al. 2013), and SIRT6 deletion upregulated toll-like receptor 4 (TLR4) and enhanced the activation of the NF-κB signaling pathway (Wu et al. 2019). Mechanistically, SIRT6 inhibits the NF-κB signaling pathway in different ways. On the one hand, SIRT6 directly mediates the deacetylation of H3K9 near the promoters of the NF-κB target genes, which significantly inhibits transcription (Liu et al. 2017; Ashburner et al. 2001). On the other hand, the NF-κB inhibitor IκBα tightly binds to NF-κB in the cytoplasm. Stress activates IκB kinase, which separates IκBα from the complex, promotes the translocation of NF-κB into the nucleus, and ultimately regulates target gene expression (Ma et al. 2014). SIRT6 induces cysteine monoubiquitination of the methyltransferase SUV39H1 leading to its removal from the IκBα gene. The consequent increase in the expression of IκBα inactivates the NF-κB pathway (Santos-Barriopedro and Vaquero 2018) (Fig. 3).

### Considering sirtuin-based strategies for reducing inflammation in IVDD

IL-10 and TGF-β inhibit the release of inflammatory factors from degenerating disc cells. IL-1β is commonly used as an inducer of disc inflammation to mimic degenerating disc tissue (Li et al. 2014a). Inhibition of nuclear translocation of NF-κB through SIRT1 deacetylation of RelA/p65 suppresses inflammation. IL-1β downregulates the expression of SIRT1 by activating TLR2, and SIRT1 inhibits IL-1β-mediated inflammatory responses through the TLR2/SIRT1/NF-κB pathway (Li et al. 2014a). Resveratrol, which acts as a SIRT1 activator, can decrease the concentrations of pro-inflammatory cytokines in vitro and has considerable potential in the treatment of myeloid cell-mediated pain (Wuertz et al. 1976).

1,4-Dihydropyridine, a novel activator of SIRT1, has antioxidant properties. It inhibits ROS-mediated inflammation and extracellular matrix degradation through the activation of SIRT1 in human NP cells (Song et al. 2020a). Monocyte chemoattractant protein 1 (MCP1) activation is involved in the initial inflammatory response associated with degenerating discs. SIRT1 inhibits MCP1 production in degenerating NP cells by inhibiting the phosphorylation of c-Fos and c-Jun factors in activator protein 1 and thus inhibits disc degeneration (Cai et al. 2020). Peroxisome proliferator-activated receptor β/δ (PPARβ/δ), which enhances IL-1β-induced COX-2 expression and PGE2 production in human thylakoid cells through the SIRT1 pathway (Li et al. 2022a). Red ginseng inhibits hypoxia-induced COX-2 expression through SIRT1 activation (Lim et al. 2015). Melatonin treatment upregulates testicular SIRT1 expression to inhibit Lipopolysaccharide (LPS)-induced inflammatory proteins, namely NF-kB/COX-2/iNOS expression (Kumar et al. 2021). It has been reported that SIRT1 inhibits activator protein-1 transcriptional activity and COX-2 expression in macrophages (Zhang et al. 2010). The regulation of COX-2 expression by SIRT1 in IVDD has not been reported yet and needs further confirmation.

Activation of the NF-κB signaling pathway promotes matrix-degrading enzyme activity in the NP and promotes extracellular matrix degradation in IVDD. SIRT6 overexpression significantly inhibits human NF-κB-dependent transcriptional activity in NP cells. Inhibition of NF-κB signaling is essential for SIRT6-mediated maintenance of extracellular matrix homeostasis in the NP (Kang et al. 2017). Moreover, 3-methyladenine and chloroquine-mediated autophagy inhibition partially reverses the anti-aging and anti-apoptotic effects of SIRT6 (Chen et al. 2018). Luteolin inhibits TNF-α-induced inflammatory damage and senescence in human NP cells through the SIRT6/NF-κB pathway (Xie et al. 2022a). Chen et al. (Chen et al. 2018) reported that IL-1β increases the levels of senescence-associated proteins (e.g., p16, p21, and p53) to promote cell death and senescence, which, in turn, reduces NP cell population and aggravates IVDD. SIRT6 overexpression downregulates the expression of senescence-associated proteins and inhibits IL-1β-induced senescence and apoptosis in NP cells.

## Sirtuins and extracellular matrix regulation

### Role of extracellular matrix in disc health and degeneration

The rates of extracellular matrix synthesis and degradation are in equilibrium in a healthy intervertebral disc. IVDD usually occurs when extracellular matrix degradation exceeds its synthesis, and type II collagen and proteoglycans are the important components lost in this process. This results in a loss of water in the disc tissue and a decrease in the cushioning and compression resistance (Emanuel et al. 2018). MMPs, the main degradative enzymes, are highly expressed in degenerated discs (Perez-Garcia et al. 2019).

### Influence of sirtuins on extracellular matrix synthesis and maintenance

SIRT maintains extracellular matrix homeostasis, regulates chondrocyte metabolism, inhibits chondrocyte apoptosis and autophagy, and prevents cellular senescence through its deacetylation activity (Sun et al. 2022) (Fig. 2). SIRT1 regulates the expression of extracellular matrix-related proteins and promotes mesenchymal stem cell differentiation. Moreover, it exerts anticatabolic, anti-inflammatory, anti-oxidative stress, and anti-apoptotic effects and participates in autophagy (Deng et al. 2019). FGF21 administration alleviated tert-butyl hydroperoxide (TBHP)-induced extracellular matrix catabolism by mediating autophagy flux through the activation of the SIRT1/mTOR signaling pathway (Lu et al. 2021). Echinacoside, the active substance of Cistanche, possesses potent anti-oxidative stress properties. It can inhibit endoplasmic reticulum stress and extracellular matrix degradation by upregulating SIRT1 in the chondrocytes of TBHP-treated mice (Lin et al. 2021b). Notably, safranal has the same effect (Liu et al. 2022). Melatonin (Zhao et al. 2022) and paeonol (Shang et al. 2022) enhanced SIRT1 expression to inactivate the NF-κB signaling pathway, which ameliorated inflammatory cytokine secretion and extracellular matrix degradation.

PEP-1–SIRT2 promote MMP-induced dedifferentiation through ERK signaling in articular chondrocytes (Eo et al. 2016). Early activation of SIRT3 in 1D sedimentation cultures significantly increased the gene expression of type II collagen, aggregated collagen, and the cartilage transcription factors SOX5 and ARID5B in extracellular matrix (Smith et al. 2022). Evodiamine upregulates SIRT3 and inhibits extracellular matrix degradation and inflammation through the activation of the PI1K/Akt pathway (Kuai and Zhang 2022). Deacetylation of FOXO1 by SIRT4 activates SOX9 expression, thereby maintaining cartilage stability in ECM (Ma et al. 2021). Circ_0022383 protects chondrocytes from IL-1β-induced apoptosis, inflammation, and degeneration through the miR-1-3619p/SIRT5 axis (Qian et al. 2022). Overexpression of SIRT6 prevents IL-1-induced NP extracellular degradation (Kang et al. 2017).

### Preserving disc integrity through sirtuin modulation

Hypoxia and a significant increase in inflammatory cytokines are common features of IVDD, and these events disrupt the normal balance between extracellular matrix degradation and synthesis in degenerative discs. SIRT1 inhibits the mRNA expression of proteases that degrade TNF-α-induced extracellular matrix (Wang et al. 2016b). SIRT1 was upregulated by tyrosol, and SIRT1 silencing inhibits the Akt phosphorylation in NP cells. SIRT1 knockdown attenuates the effect of tyrosol on IL-1β-induced apoptosis, inflammation, and extracellular matrix remodeling in NP cells (Qi et al. 2020). Overexpression of SIRT1 mediated by lentiviral vectors inhibits IL-1β-induced extracellular matrix degradation and apoptosis. In contrast, siRNA knockdown of the gene encoding SIRT1 increases IL-1β-induced MMP expression and apoptosis (Shen et al. 2016). Orientin downregulates oxidative stress-mediated endoplasmic reticulum stress and mitochondrial dysfunction through the AMPK/SIRT1 pathway in rat NP and attenuates disc degeneration (Zhang et al. 2022). Hyperoside upregulates SIRT1 and nuclear factor E2-related factor 2 (Nrf2) protein expression and ameliorates TNF-α-induced inflammation and extracellular matrix degradation (Xie et al. 2022b).

The activation of the AMPK/PGC-1α pathway partially alleviates oxidative stress, senescence, and degeneration induced by the knockdown of SIRT3 in NP cells (Lin et al. 2021a). SIRT6 prevents the degradation of the NP extracellular matrix in vitro by inhibiting the NF-κB-dependent transcriptional activity, thereby ameliorating disc degeneration (Kang et al. 2017) (Fig. 2).

## Regulation of apoptosis by sirtuins in IVDD

### Apoptosis contributes to cell death and degeneration in intervertebral discs

Apoptosis refers to the genetically controlled, autonomous, and orderly death of cells to maintain the stability of the internal environment. Apoptosis, also known as type I programmed death, is involved in many physiologic processes, such as growth, development, and prevention of malignant transformation of cells. The reduction in the number of cells in the intervertebral disc due to apoptosis and a decrease in extracellular matrix synthesis and alteration of its composition are the key features of disc degeneration (Fine et al. 2023).

### Anti-apoptotic effects of SIRT1 and its potential relevance

SIRT1 regulates many important genes through deacetylation, such as p53, FOXO, PGC-1α, and protein kinase B (PKB/Akt). These downstream genes, in turn, regulate disc cell apoptosis by modulating apoptotic factors, neuroinflammation, oxidative stress, and mitochondrial development.

P53 is a tumor suppressor protein that belongs to the family of p63 and p73. P53 is a key component of the cellular stress response. The protein is regulated by modulating its gene expression and stability, as well as various reversible post-translational modifications. P53 is induced or undergoes rapid reversal of post-translational modifications for stabilization and activation in response to DNA damage, oncogene expression, hypoxia, increased ROS, and nutrient deficiencies. P53 transcriptional activation in the nucleus is involved in apoptosis, cell cycle, autophagy, and metabolism (Kruiswijk et al. 2015). In contrast, cytoplasmic p53 acts in a transcription-independent manner and directly binds to cytoplasmic apoptosis and autophagy effectors (Saldana-Meyer and Recillas-Targa 2011). P53 mainly interacts with the Bcl family proteins, including the anti-apoptotic protein Bcl-2, which induces Bak oligomerization, permeabilizes mitochondrial membranes, induces cytochrome C release, and activates apoptotic protease cascades, thereby regulating apoptosis. Several lysine residues of p53, including K320, K373, and K382, can be acetylated in vivo. The acetylation of different residues induces different aspects of the p53-mediated stress response. Acetylation of the K382 residue activates p53 to trigger the transcription of apoptosis-associated target genes (Wang et al. 2014). SIRT1 deacetylates the lysine residue at the K382 position to reduce the transcriptional activity of p53 and inhibit apoptosis (Li et al. 2014b). P53 is ubiquitinated at the C-terminal position, and SIRT1 deacetylates these residues during cellular stress to block proteolytic degradation and ensure stabilization of p53 (Liu et al. 2018a).

FOXO1 and FOXO3 are two of the most widely studied isoforms of FOXO transcription factors, which are involved in the pathogenesis of several diseases and stem cell activity (Liu et al. 2018a). FOXO1/3 upregulates several cell cycle inhibitors and pro-apoptotic targets and plays important roles in cell proliferation, differentiation, and apoptosis in the heart, vasculature, skeletal muscle, liver, and brain (Zhang et al. 2017). Transcriptional activation of FOXO can be regulated by acetylation/deacetylation or phosphorylation/dephosphorylation. Lysine residues in the DNA-binding region of the FOXO protein can be acetylated by cytosolic proteins with histone acetyltransferase activity, decreasing its transcriptional activation. The FOXO family of proteins is a common target of SIRT1, essential for mediating apoptosis. SIRT1 has a dual effect on the function of FOXO3a. It enhances the ability of FOXO3a to promote cell cycle arrest and resistance to oxidative stress and inhibits its ability to induce cell death through SIRT1-mediated deacetylation (Brunet et al. 2004).

PGC-1α is a nuclear transcriptional co-activator of nuclear receptors and several other transcription factors. PGC-1α is involved in the positive regulation of oxidative metabolism and counteracts ROS to enhance cellular antioxidant capacity (Khan et al. 2012). Histone acetyltransferase complex directly acetylates multiple lysine residues of PGC-1α, leading to a decrease in its levels and transcriptional activity. In contrast, SIRT1 deacetylates PGC-1α to enhance its activity, promote mitochondrial biosynthesis, and maintain mitochondrial function to reduce apoptosis (Zhou et al. 2017).

Akt is a serine-threonine kinase activated by phosphorylated phosphatidyl kinase-3-kinase products. The movement of Akt from the cytoplasm to the plasma membrane induces the activation of the kinase and regulates the phosphorylation of Akt substrates, ultimately influencing various physiologic and pathologic processes. Expression of SIRT1 increases Akt activity and inhibits apoptosis (Iaconelli et al. 2017). Histone acetyltransferases acetylate Akt at Lys-14 and Lys-20; phosphorylation of acetylated Akt decreases, inhibiting its activity. In contrast, SIRT1 deacetylates Akt at the same positions to promote its activation. Activated Akt phosphorylates its substrates, such as Bad, FOXO, and glycogen synthase kinase 3β to inhibit apoptosis (Li et al. 2018a). The non-phosphorylated Bad translocates to mitochondria and triggers cytochrome C release, cystatinase-3 activation, and apoptosis in the absence of activated Akt. Non-phosphorylated FOXO proteins translocate to the nucleus and act as transcription factors to increase the protein levels of Bim and Fas ligands. Bim also triggers cytochrome C release, whereas Fas triggers the extrinsic apoptotic pathway (Iaconelli et al. 2017) (Fig. 2).

### Exploring avenues for enhancing cell survival through sirtuin modulation

CircRNAs and miRNAs are the intracellular regulators that modulate iodine deficiency disorders (Yang et al. 2021). miR-34a-5p binds to SIRT1 mRNA, and its overexpression inhibits miR-34a-5p-induced cell cycle arrest and senescence (Zhu et al. 2021). CircRNA-CIDN acts as a miR-34a-5p sponge to inhibit compression-induced apoptosis and extracellular matrix degradation in NP. The combination of CircRNA-CIDN and miR-34a-5p diminishes compressive loading-induced myeloid cell injury by targeting SIRT1 (Xiang et al. 2020). Xie et al. (2019) demonstrated that circERCC2 promotes filamentophagy response by regulating apoptosis, mitosis, and extracellular matrix degradation in NP and decreases apoptosis and extracellular matrix degradation to alleviate IVDD. In contrast, circRNA_0000253 is highly upregulated in degenerate myeloid exosomes and can promote IVDD by adsorbing miRNA-141-5p and downregulating SIRT1 (Song et al. 2020b).

Overexpression of miR-199a-5p promotes apoptosis and IVDD in myeloid cells. The effects of miR-199a-5p overexpression were diminished upon SIRT1 overexpression, whereas its silencing diminished the effects of p21 overexpression. Furthermore, miR-199a-5p promoted myeloid apoptosis and IVDD by inhibiting SIRT1-dependent p21 deacetylation (Sun et al. 2021a) (Fig. 2). The downregulation of miR-138-5p upregulated SIRT1 expression by directly targeting its 3'-untranslated region, and mutations in the miR-138-5p binding site inhibited this effect. The inhibition of miR-138-5p decreased the PTEN protein expression and promoted the PI3K/AKT activation. In contrast, inhibition of SIRT1 or PI3K/AKT inhibitor treatment eliminated the effect of miR-138-5p on NP cell apoptosis. miR-138-5p knockdown protects human NP cells from apoptosis by upregulating SIRT1, and this effect may be mediated through the PTEN/PI3K/Akt signaling pathway (Wang et al. 2016c). miR-22-3p protects human NP cells from apoptosis by targeting SIRT1 and plays a mechanistic role in the development of IVDD. SIRT1, in turn, activates the JAK3/STAT22 signaling pathway. Delivery of miR-22-3p inhibitors and mimics through nanoparticles synthesized in the IVDD model alleviated and exacerbated IVDD, respectively. The nanocarriers enhanced miR-22-3p translocation to myeloid NP cells, which led to in vivo inhibition of miR-22-3p, thereby facilitating the development of miRNA-specific drugs for IVDD (Chen et al. 2023a). miR-141 promotes IVDD by targeting and depleting SIRT1, a negative regulator of the NF-κB pathway (Ji et al. 2018). miR-106b-5p overexpression decreases cell growth, induces apoptosis, impairs extracellular matrix formation, and increases the expression of matrix-degrading enzymes in NP cells through the SIRT2/MAPK/ERK signaling pathway (Meng et al. 2023).

NP mesenchymal stem cells (NPMSCs) in the intervertebral disc can promote regeneration due to their endogenous repair function and differentiation potential (Liao et al. 2019). Liao et al. (2019) demonstrated that overexpression of SIRT1 diminishes IVDD by inactivating the MCP1/chemokine receptor 2 axis, which promotes chondrogenic differentiation and reduces apoptosis of NPMSCs. Liu et al. (2020a) demonstrated that high-glucose culture significantly decreased the stem cell gene expression and the mRNA and protein expression of SIRT1, SIRT6, HIF-1α, and glucose transporter protein type 1, while increasing apoptosis, senescence, and caspase-3 expression in NPMSCs. The high sugar concentration significantly decreased the cell proliferation, colony-forming ability, migration, and wound-healing ability of NPMSCs. Notably, NPMSCs cultured in high glucose concentrations had a significantly low expression of stemness gene-related mRNAs and proteins.

## Oxidative stress management by sirtuins

### Effect of oxidative stress on IVDD development

Oxidative stress is a pathologic condition characterized by an imbalance between the generation of ROS and the corresponding antioxidant system (Xian et al. 2021). ROS are incompletely reduced oxygenated molecules with strong reactivity, which can diffuse in the cells to destroy nucleic acids, proteins, lipids, and other molecules (Cheung and Vousden 2022). The human body has an antioxidant system that scavenges ROS, and a homeostasis of oxidative and antioxidant systems exist under normal conditions. However, excessive ROS production or decline in the function of the antioxidant system leads to an imbalance, i.e., oxidative stress, which ultimately destroys critical molecules or cells in the body (Endale et al. 2023). The accumulation of cells damaged by ROS activity further accelerates the production of ROS, forming a vicious cycle.

ROS are extensively involved in signaling, metabolic regulation, programmed cell death, senescence, and phenotypic transformation of intervertebral disc cells. Notably, IVDD is a disc cell-mediated pathologic process, and disc degeneration is closely related to the viability and function of disc cells (Li et al. 2022b). Oxidative stress induced by excessive ROS activates multiple signaling pathways in disc cells, prompting a phenotypic shift from a matrix anabolic phenotype to a catabolic and pro-inflammatory phenotype, leading to matrix loss and enhanced inflammation in the disc microenvironment. In addition, chemokines secreted by disc cells recruit more immune cells to the disc, further exacerbating inflammation. These immune cells secrete more cytokines and chemokines, which again reduce the viability and function of the disc cells (Risbud and Shapiro 2014).

Oxidative stress is a potent trigger of autophagy, apoptosis, and senescence in NP cells. Autophagy protects NP cells from oxidative damage by providing metabolic substrates for recycling under oxidative stress. However, excessive autophagy induced by persistent oxidative stress will lead to autophagic death of NP cells (Zheng et al. 2019). Moreover, oxidative stress directly induces apoptosis in NP cells. Therefore, the activity and function of NP cells are significantly reduced under sustained oxidative stress, and this reduction cannot be compensated by cell proliferation due to NP cell senescence. Moreover, senescent NP cells secrete pro-inflammatory cytokines that promote the death or senescence of neighboring NP cells, further reducing the count of viable and functional cells (Chen et al. 2019).

### Role of sirtuins in enhancing antioxidant defenses

**Fig. 4:**
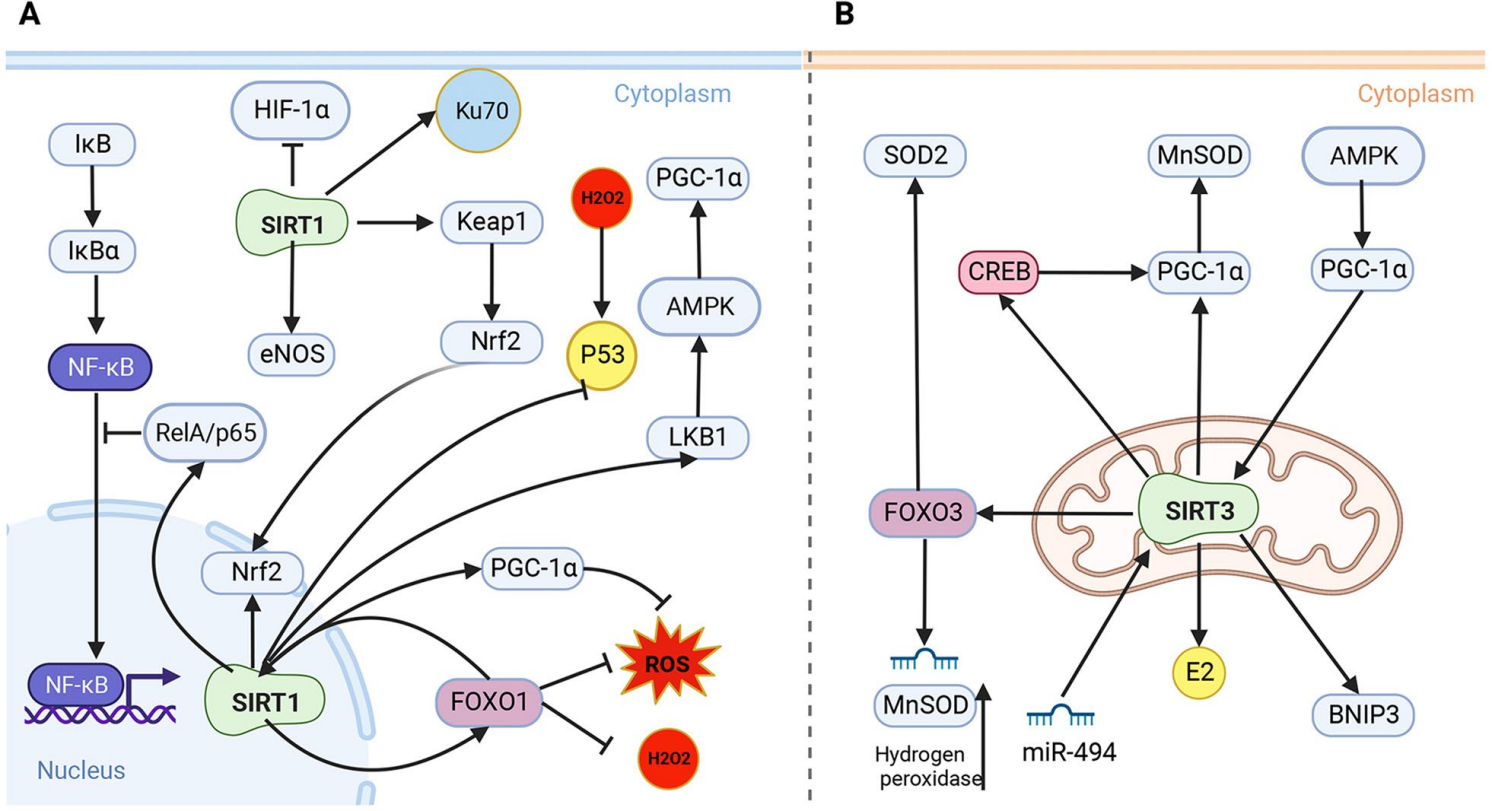
SIRT is associated with the management of oxidative stress associated with IVDD. **A** SIRT1 can regulate a variety of target genes and target proteins, for example, SIRT1 can activate Nrf2, FOXO1, PGC-1α, LKB1, and RelA/p65, and SIRT1 can inhibit p53 and HIF-1α, etc., and thus plays an important role in oxidative stress injury. **B** SIRT3 is associated with the management of oxidative stress associated with IVDD.SIRT3 regulates the expression of antioxidant enzymes through activating FOXO3, PGC-1α, E2, and CREB.SIRT3 is associated with the management of oxidative stress associated with IVDD. SIRT3 is associated with the management of oxidative stress associated with IVDD.SIRT3 regulates the expression of antioxidant enzymes through the activation of FOXO3, PGC-1α, E2 and CREB

SIRT1 regulate NF-κB (Li et al. 2018b), FOXO1 (Yao et al. 2018), P53 (Lin et al. 2018b), PGC-1α (Tang 2016), AMPK (Wang et al. 2019b), Nrf2 (Tang et al. 2014), HIF-1α (Shin and Lee 2017), endothelial nitric oxide synthase (eNOS) (Ding et al. 2015), Ku70 (Jeong et al. 2007), and other target genes/ proteins, which play an important role in oxidative stress injury(Fig. 4A). Activated NF-κB factors activate inflammatory mediators that damage the body and simultaneously promote ROS production, which damages tissues and organs and further promotes the expression of inflammatory factors. NF-κB binds to its inhibitor IκBα in the cytoplasm to form an inactive complex under normal conditions. IκB kinase phosphorylates IκBα and releases NF-κB upon stimulation, which then enters the nucleus and binds to the corresponding promoter gene target to enhance transcription. SIRT1 deacetylates RelA/p65, binds NF-κB to IκBα, and inhibits NF-κB transcriptional activity, thereby reducing the expression of inflammatory factors (Li et al. 2018b).

FOXO1 can scavenge excessive ROS by regulating downstream target genes, such as MnSOD and catalase, thereby reducing cellular oxidative stress damage (Meng et al. 2017). SIRT1 activates FOXO1 through deacetylation, attenuates H_2_O_2_-induced cellular oxidative stress injury, and inhibits osteoblast apoptosis. Xiong et al. (2011) reported that FOXO1 increases the expression level of SIRT1. This suggests that a feedback mechanism may be involved in FOXO1-dependent SIRT1 transcription and SIRT1-mediated deacetylation of FOXO1.

P53 promotes oxidative stress damage and induces apoptosis by regulating different target proteins, such as P53-inducible protein, reduced nicotinamide adenine dinucleotide phosphate cytoplasmic subunit NCF2/p67phox, p66Shc, and Bax (Liu et al. 2020b). The H_2_O_2_-induced oxidative stress environment increases the expression and accumulation of the p53 gene, whereas activation of SIRT1 decreases its activity (Kim et al. 2015). Lin et al. (2018b) confirmed that SIRT1 can protect renal tubular cells from oxidative stress injury and reduce apoptosis by deacetylating p53 and inhibiting its activation. Overall, SIRT1 can inhibit p53 activation through deacetylation and apoptosis, thereby resisting oxidative stress injury.

PGC-1α may play an anti-oxidative role by scavenging excessive ROS, inducing the expression of antioxidant enzymes, and maintaining mitochondrial function (Iacovelli et al. 2016). SIRT1 can regulate metabolic disorders through deacetylation of PGC-1α and decrease cellular damage caused by external stimuli (Tang 2016). Liang et al. (2020) showed that SIRT1 activates PGC-1α through deacetylation, scavenges ROS generated by oxidative stress, and attenuates intestinal oxidative stress injury. Therefore, SIRT1 can activate the expression of PGC-1α through deacetylation and ameliorate oxidative stress injury.

AMPK can be activated by liverkinase B1, and activated AMPK ameliorates oxidative stress injury by promoting insulin sensitivity, fatty acid oxidation, and mitochondrial biosynthesis to produce ATP. Overexpression of SIRT1 can lead to deacetylation of liverkinase B1 and changes its localization from the nucleus to the cytoplasm, where it activates AMPK (Vancura et al. 2018). Wang et al. (2019b) showed that SRT1720 (a SIRT1 agonist) increased the expression of AMPK and improved the antioxidant capacity in type 2 diabetic rats. In addition, SIRT1 and AMPK can regulate each other, and both of them can directly affect the activity of PGC-1α through acetylation and phosphorylation, respectively. This series of events eventually ameliorates the oxidative stress injury (Canto et al. 2009).

Nrf2 is a leucine transcription factor composed of six structural domains NRF2 ECH homology 1–6. It plays a crucial role in the transcriptional regulation of antioxidant response element (ARE)-dependent defense genes. Nrf2 binds to the inhibitory protein Keap1 in the cytoplasm and exists as an inactive Nrf2–Keap1 complex. Nrf2 dissociates from Keap1 and enters the nucleus upon stimulation, where it interacts with ARE and regulates the expression of antioxidant genes, such as glutathione S-transferase, glucuronosyltransferase, and heme oxygenase (Zhang et al. 2013). SIRT1 can activate Nrf2 by modifying the structure of Keap1, leading to nuclear translocation of Nrf2, thereby promoting the expression of antioxidant genes (Tang et al. 2014). Huang et al. (2015) found that resveratrol directly promotes the deacetylation and subsequent activation of Nrf2 by increasing the expression of SIRT1, which ultimately upregulates the expression of target genes of Nrf2 (antioxidant genes). Chen et al. (2023b) showed that populin may exert a protective effect against ischemic encephalopathy injury by alleviating oxidative stress and apoptosis through the NRF2 signaling pathway. And it has been demonstrated that populin can prevent intervertebral disc degeneration in rats through the Nrf2/HO-1/NF-κB signaling pathway (Mao and Fan 2023). Xie et al. (2023) found that populin affected the SIRT1/PGC-1α pathway to protect mitochondrial function and alleviate cellular senescence in H_2_O_2_-treated myeloid MSCs. Overall, SIRT1 can directly or indirectly activate Nrf2 and regulate the expression of antioxidant genes to protect the cells from oxidative stress damage.

The HIF family consists of HIF1, HIF2, and HIF3 proteins. HIF expression increases during oxidative stress and inflammation in hypoxic tissues. HIF regulates various genes, such as erythropoietin, vascular endothelial growth factor, and glycolytic enzymes, which play an important role in vascularization, energy metabolism, cell survival, apoptosis, and the maintenance of cellular stability under hypoxic conditions (Choudhry and Harris 2018). The activation of HIF-1α is associated with oxidative stress and can directly and indirectly regulate ROS formation (Stuart et al. 2019). Shin et al. (2017) found that an increase in SIRT1 decreases the HIF-1α acetylation level. In contrast, the knock down of SIRT1 significantly increases the HIF-1α acetylation level, indicating that SIRT1 regulates HIF-1α acetylation to resist hepatic ischemia–reperfusion injury. Therefore, SIRT1 may play a regulatory role in oxidative stress by modulating HIF-1α expression.

eNOS is mainly expressed in endothelial cells and plays an important role in cardiac and vascular oxidative stress. SIRT1 plays an important role in regulating eNOS activity, and upregulation of SIRT1 can decrease eNOS acetylation (inactivated state) and increase its phosphorylation (activated state) (Ding et al. 2015). Moreover, activation of the SIRT1/eNOS pathway inhibits oxidative stress and ameliorates ischemia–reperfusion injury in the relevant animal models. Ku70 is a DNA repair protein involved in DNA damage repair and apoptosis caused by various stimuli. All types of oxidative stress damage may cause DNA damage. SIRT1 enhanced DNA repair activity after radiation exposure by deacetylating Ku70. The pro-apoptotic protein Bax binds to Ku70 under physiologic conditions. Ku70 is acetylated upon oxidation stimulation and dissociates from Bax, and the dissociated Bax is then localized to the mitochondrial membrane to promote cell apoptosis (Jeong et al. 2007). SIRT1 can deacetylate Ku70, which strengthens its interaction with Bax. Consequently, the transfer of Bax to the mitochondrial membrane is inhibited, thereby inhibiting apoptosis in rat germ cells and ultimately alleviating cellular oxidative stress injury (Liu et al. 2018b).

Several authors have evaluated the role of SIRT3 in oxidative stress (Fig. 4B). SIRT3 regulates the expression of antioxidant enzymes through FOXO3a and PGC-1α. Jocobs et al. reported for the first time that SIRT3 interacts with FOXO3a to form a complex that regulates the activity of FOXO3a. This interaction enhances the binding of FOXO3a to both promoters, altering the intracellular oxidative environment (Jacobs et al. 2008). Activation of FOXO3a by SIRT3 increased the levels of mRNA transcripts of several antioxidant genes, including MnSOD and catalase genes, consequently increasing the levels of corresponding proteins (Sundaresan et al. 2009). The deacetylation of FOXO3a by SIRT3 protects mitochondria from oxidative stress by modulating FOXO3a-dependent expression of the antioxidant genes, which is associated with the intervention of aging-related pathogenesis (Tseng et al. 2013).

SIRT3 regulates the transcriptional regulator PGC-1α to upregulate MnSOD expression. PGC-1α further regulates the expression of several mitochondrial antioxidant genes, including MnSOD and catalase (Valle et al. 2005). Transcriptional activation of PGC-1α is mainly regulated by CREB in different tissues (Lopez-Lluch et al. 2008). SIRT3 promotes the phosphorylation of CREB, which, in turn, triggers the transcriptional activation of PGC-1α (Rius-Perez et al. 2020). In addition, activation of SIRT3 in a pressure-overload hypertrophic mouse model increases the levels of PGC-1α mRNA, resulting in reduced oxidative stress and improved mitochondrial function (Pillai et al. 2015). However, PGC-1α deletion reduces SIRT3 gene expression in myocytes and hepatocytes, which is mediated by estrogen-related receptor-binding element (Kong et al. 2010). These results suggest a bidirectional regulation between SIRT3 and PGC-1α involved in mitochondrial antioxidant defense.

### Sirtuin-based strategies for mitigating oxidative damage

Oxidative stress-induced senescence of NP cells is an important cause of IVDD. Regarding SIRT 1 affecting IDD action through oxidative stress, we have discussed it in detail in the aging section. Here, we mainly reviewed the effect of SIRT3 on IVDD. Dysregulation of SIRT3 and secondary imbalance of mitochondrial redox homeostasis are important mechanisms mediating oxidative stress-induced senescence and apoptosis in NP cells. SIRT3 expression was downregulated with the progression of human IVDD. In vitro silencing of SIRT3 reduced cellular resistance to oxidative stress and upregulated senescence and apoptosis in rat NP cells. In contrast, activation of SIRT3 significantly inhibited oxidative stress-induced senescence and apoptosis of NP cells and delayed IVDD (Wang et al. 2018a). Similar effects of SIRT3 on oxidative stress-induced senescence of NP cells have been reported in vitro (Lin et al. 2021a). Some authors used H_2_O_2_ to induce oxidative stress in NP cells, and H_2_O_2_ treatment upregulated the expression of SIRT3 in NP cells. The upregulated SIRT3 exerted cytoprotective effects. Therefore, SIRT3 upregulates the activity of the mitochondrial antioxidant enzyme system, scavenges intracellular ROS, and maintains cellular homeostasis and physiological functions (Wang et al. 2018a; Lin et al. 2021a). The deubiquitinating enzyme USP11 stabilizes SIRT3 by directly binding to and deubiquitinating SIRT3. Therefore, USP11 overexpression significantly ameliorates oxidative stress-induced iron-dependent cell death and alleviates IVDD through SIRT3 upregulation (Zhu et al. 2023).

Nicotinamide mononucleotide attenuates oxidative stress-induced apoptosis in NP cells by attenuating the inhibitory effect of AGEs on SIRT3 (Song et al. 2018). The activation of the AMPK/PGC-1α pathway enhanced the cytoprotective effect of SIRT3, whereas inhibition of this pathway with compound C attenuated the cytoprotective effect of SIRT3 on NP cells (Wang et al. 2018a; Song et al. 2018). SIRT3 can upregulate SOD2 expression by activating FOXO3a, thereby exerting an antioxidant effect and delaying the degenerative process in intervertebral disc (Zhou et al. 2019). Recently, Hu et al. reported that SIRT3 inhibits oxidative damage and apoptosis in NP cells by activating downstream mitochondrial autophagy to promote Nrf2-mediated antioxidant effects (Hu et al. 2021).

SIRT3 activation promotes mitochondrial autophagy in NP cells, thereby attenuating oxidative stress-induced senescence and apoptosis. In addition, SIRT3 activates mitochondrial autophagy through a BNIP3-dependent pathway after anti-staphylococcal treatment (Wang et al. 2018a). miR-494/SIRT3/mitochondrial autophagy signaling pathway promotes apoptosis and mitochondrial dysfunction in NP cells. Duhuo Jisheng Decoction protects from IVDD by regulating this signaling axis (Liu et al. 2023). Wang et al. (2019c) demonstrated that metformin protects primary chondrocytes from PINK1/Parkin-mediated mitochondrial autophagy through SIRT3 activation. However, the regulatory network of autophagy is complex. Therefore, further studies are needed to assess whether SIRT3 is associated with macroautophagy and the PINK1/Parkin-dependent classical mitochondrial autophagy pathway in intervertebral disc cells.

### Therapeutic implications and future directions

#### Potential of sirtuin-targeted interventions in IVDD

Resveratrol is a phenolic derivative mainly found in grape skins and seeds (Bhat et al. 2001). This compound exerts anti-inflammatory, antioxidant, and aging-delaying effects in cells and can be used for treating chronic diseases, such as diabetes, obesity, cardiovascular disease, and cancer (Malaguarnera 2019; Galiniak et al. 2019). Resveratrol can reduce apoptosis and senescence of NP cells and promote the synthesis of proteoglycans in the extracellular matrix, thereby slowing down the process of IVDD (Lin et al. 2018a). Moreover, resveratrol can activate downstream signaling molecules including SIRTs (Wang et al. 2018b; Gomes et al. 2018). Therefore, resveratrol is widely used as an activator of SIRTs in several experiments.

Honokiol (C18H18O2) is a naturally occurring small molecule compound extracted from the roots and bark of *Magnolia officinalis*. It possesses various pharmacologic properties, including anti-inflammatory, antioxidant, analgesic, and neuroprotective effects (Lin et al. 2007; Tang et al. 2018). Notably, honokiol mediates an increase in SIRT3 activity (Kanwal 2018; Zheng et al. 2018). Wang et al. (2018a) reported that honokiol enhanced mitochondrial antioxidant capacity, mitochondrial dynamics, and mitochondrial function upon SIRT3 activation through the AMPK/PGC-1α signaling pathway, thereby rescuing oxidative stress-induced NP cells from apoptosis and senescence. Moreover, honokiol significantly ameliorated IVDD in a rat model. Naringin enhances autophagic flow through AMPK activation and SIRT1 upregulation, thereby protecting NP cells against inflammatory responses, oxidative stress, and impaired cellular homeostasis (Chen et al. 2022). Quercetin inhibits apoptosis and ameliorates IVDD through the SIRT1/autophagy axis (Wang et al. 2020b).

Melatonin is an endocrine hormone synthesized and secreted by the pineal gland in the brain and plays an important role in the maintenance of circadian rhythms (Zisapel 2018). Melatonin treatment reduces apoptosis and inhibits endplate chondrocyte calcification in a dose-dependent manner. In addition, melatonin upregulates SIRT1 expression and activity and promotes autophagy in endothelial progenitor cells. However, inhibition of autophagy using 3-methyladenine reverses the protective effects of melatonin on apoptosis and calcification. The SIRT1 inhibitor EX-527 inhibited melatonin-induced autophagy and the protective effects of melatonin on apoptosis and calcification. These findings suggest that the beneficial effects of melatonin are mediated through the SIRT1/autophagy pathway (Zhang et al. 2019b). Melatonin inhibits M1-type macrophage polarization and ameliorates inflammation-induced NP cell injury through the SIRT1/gap signaling pathway, which is important for the remission of IVDD (Dou et al. 2023).

Nimbolide, a natural compound isolated from *Azadirachta indica*, activates SIRT1 in NP cells during inflammation to promote cholesterol efflux and inhibit the activation of NF-κB and MAPK signaling pathways, which balance matrix anabolism and catabolism. However, SIRT1 inhibition significantly diminishes the effects of nimbolide. Furthermore, nimbolide promoted SIRT1 expression in RAW 264.7 cells, increased the proportion of M2 macrophages by promoting cholesterol homeostatic reprogramming, and impaired M1-like macrophage polarization by blocking the activation of inflammatory signals. Therefore, nimbolide can be potentially used for the treatment of IVDD (Teng et al. 2023).

Small extracellular vesicles released from induced pluripotent stem cell-derived mesenchymal stem cells (iMSC-sEVs) can restore NP cell senescence and slow down the progression of IVDD. iMSC-sEVs exert their anti-aging effects by delivering miR-105-5p to senescent NP cells and activating the SIRT6 pathway. Sun et al. (2021b) showed that iMSCs are a promising candidate for obtaining sEVs on a large scale while avoiding some of the pitfalls associated with the current applications of MSCs. Therefore, iMSC-sEVs may be a novel cell-free therapeutic tool for the treatment of IVDD. Although iMSC-sEVs are currently the main research direction in nanomedicine, a current study found that Platelet-derived extracellular vesicles (PEVs) can improve IVDD by improving mitochondrial function. PEVs can restore impaired mitochondrial function by modulating the sirtuin 1 (SIRT1)-peroxisome proliferator activated receptor γ coactivator 1α (PGC1α)-mitochondrial transcription factor A (TFAM) pathway to reduce oxidative stress. body proliferator-activated receptor γ coactivator 1α (PGC1α)-mitochondrial transcription factor A (TFAM) pathway to restore impaired mitochondrial function, reduce oxidative stress, and restore cellular metabolism; PEVs delayed the progression of IVDD in a rat model (Dai et al. 2023). Thus, this nanomaterial therapy may offer more potential therapeutic prospects in the future.

### Challenges and limitations in translating basic research on sirtuins to therapies

Although some authors have reported the regulation of myeloid cells by SIRTs, studies on ex-SIRT1 are limited. Therefore, further studies are needed to assess their mechanisms of action. In addition, there are no effective drugs for the treatment of iodine deficiency disorders. Several authors have examined SIRT-mediated regulation of autophagy and apoptosis in myeloid cells but not in cartilage endplate cells. Therefore, basic and clinical studies should be conducted on the use of SIRTs for the diagnosis and treatment of IVDD. Studies have been conducted using simple and stable animal models of IVDD, such as the rat caudal pinning model. However, the stress response in the rat caudal spine is different from that in the human IVDD. Therefore, this animal model does not fully mimic the pathologic process of human IVDD. Further, the role of SIRTs in the pathogenesis of IVDD has not been evaluated using transgenic animals. Most in vivo studies have been performed using drug-induced models having poor specificity. Therefore, the use of transgenic animal models can provide reliable evidence for the role of SIRTs in the pathogenesis of IVDD.

### Identifying areas for future research and clinical investigations

Compounds, such as resveratrol, honokiol, melatonin, naringin, and quercetin have therapeutic effects on IVDD. However, most of these compounds have been evaluated in only preclinical studies. Animal studies and preliminary clinical trials are needed to further validate the efficacy and safety of these compounds. In addition, the expression of SIRTs is reduced in senescent MSCs, and knockdown of SIRTs leads to an accelerated senescence phenotype in MSCs accompanied by abnormal mitochondrial function. However, abnormal mitochondrial function and delayed cellular senescence can be partially ameliorated by backfilling with SIRTs or overexpression of SIRTs in old MSCs (Diao et al. 2021). Stem cell therapy has been used for the clinical treatment of degenerated intervertebral discs (Garcia-Sancho et al. 2017; Pettine et al. 2017). Given that myeloid stem cells also exist in the NP and can repair degenerated myeloid tissues (Liao et al. 2019; He et al. 2021b), new therapeutic avenues can be explored if the aging of myeloid stem cells is closely related to SIRTs (Table 3).

**Table 3:** Therapeutic implications and future directions

Authors (reference)	Type of study	Study design	Aim	Results	Conclusion
Wang et al. (2018b)	An experimental study	Experimental in vitro study	To investigate the whether resveratrol can protect against high glucose-induced NP cell apoptosis and senescence, and the potential mechanism in this process	High sugar significantly promoted NP cell apoptosis and NP cell senescence. Resveratrol was protective against high glucose-induced NP cell apoptosis and senescence. Resveratrol inhibited the production of reactive oxygen species (ROS) and increased the activity of the PI3K/Akt pathway	Resveratrol can attenuate high glucose-induced NP cell apoptosis and senescence, and the activation of ROS-mediated PI3K/Akt pathway may be the potential mechanism in this process
Lin et al. (2007)	An experimental study	Experimental in vivo study	The effects of Honokiol and magnolol on formalin-induced c-Fos expression in the dorsal horn of the spinal cord, as well as motor coordination and cognitive function, were investigated	Honokiol and magnolol significantly reduced formalin-induced c-Fos protein expression in the superficial (I-II) layer of the L4-L5 lumbar dorsal horn. and thujaplicin and xylenol did not cause motor incoordination and memory dysfunction at doses higher than the analgesic dose	Honokiol and magnolol effectively alleviate the formalin-induced inflammatory pain without motor and cognitive side effects
Tang et al. (2018)	An experimental study	Experimental in vitro study	The present study was undertaken to examine the antiinflammatory, antioxidation and IVD-protective effect of honokiol using nucleus pulposus cells and investigate its mechanisms to provide a new basis for future clinical treatment of IVDD	Honokiol inhibits the H2O2-induced apoptosis (caspase-9, caspase-3, and bax), levels of oxidative stress mediators (ROS, MDA), expression of inflammatory mediators (Interleukin-6, COX-2, and iNOS), major matrix degrading proteases (MMP-3, MMP-13, ADAMTS5, and ADAMTS4) associated with nucleus pulposus degradation	Honokiol inhibited the H2O2 induced apoptosis, oxidative stress, and inflammatory responses through the depression of TXNIP/NLRP3/caspase-1/ Interleukin—1β signaling axis and the activation of NF-kB and JNK
Zheng et al. (2018)	An experimental study	Experimental in vivo and in vitro study	This study was designed to investigate the mechanisms of neuroprotection of sirt3 in hyperglycemic ICH	Hyperglycemia after ICH inhibits sirt3 expression. Hyperglycemic ICH induces extensive mitochondrial vacuolization. hkl attenuates ROS accumulation via the Sirt3-superoxide dismutase 2 (SOD2) and Sirt3-NRF1-TFAM pathways. sirt3 activation reduces NLRP3 and interleukin-1β levels by deacetylating SOD2 and scavenging ROS	HKL protects against hyperglycemic ICH-induced neuronal injury via a sirt3-dependent manner
Chen et al. (2022)	An experimental study	Experimental in vitro study	The goal of this study was to explore particular autophagic signalings responsible for the protective effects of naringin, a known autophagy activator, on human NP cells	Significantly increased autophagic flux was observed in NP cells treated with naringin, with pronounced decreases in the inflammatory response and oxidative stress, which rescued the disturbed cellular homeostasis induced by TNF-α activation	Naringin boosts autophagic flux through SIRT1 upregulation via AMPK activation, thus protecting NP cells against inflammatory response, oxidative stress, and impaired cellular homeostasis
Wang et al. (2020b)	An experimental study	Experimental in vivo and in vitro study	To investigate the specific therapeutic effects of quercetin on IDD	Quercetin treatment inhibited the apoptosis of NP cells and ECM degeneration induced by oxidative stress.quercetin promoted the expression of SIRT1 and autophagy in NP cells in a dose-dependent manner. Autophagy inhibitor 3-methyladenine (3-MA) reversed the protective effect of quercetin on apoptosis and ECM degeneration	Quercetin prevents IDD by promoting SIRT1-dependent autophagy
Zhang et al. (2019b)	An experimental study	Experimental in vitro study	The effects of melatonin on EPC apoptosis and calcification and elucidated the underlying mechanism	Melatonin treatment decreases the incidence of apoptosis and inhibits EPC calcification in a dose-dependent manner	Melatonin reduces EPC apoptosis and calcification and that the underlying mechanism may be related to Sirt1-autophagy pathway regulation
Dou et al. (2023)	An experimental study	Experimental in vivo and in vitro study	The study aims to explore the medical prospect of melatonin (MLT) and the underlying therapeutic mechanism of MLT-mediated macrophage (Mφ) polarization on the function of nucleus pulposus (NP) in intervertebral disc degeneration (IDD)	Inhibition of SIRT1 and enhancement of Notch were observed in activated Mφs and could be reversed after MLT treatment	MLT inhibits M1-type Mφ polarization and ameliorates inflammation-induced NP cell damage both in vitro and in vivo
Teng et al. (2023)	An experimental study	Experimental in vivo and in vitro study	To explore whether Nimbolide (Nim) can alleviate IDD	Nim promotes cholesterol efflux and inhibits activation of nuclear factor kappa B (NF-κB) and mitogen-activated protein kinase (MAPK) signaling pathways through activation of sirtuin 1 (SIRT1) in nucleus pulposus cells (NPC) during inflammation	Nim may represent a new therapeutic strategy for the treatment of IDD
Sun et al. (2021b)	An experimental study	Experimental in vivo study	This study aimed to investigate the therapeutic effect of sEVs derived from iMSC (iMSC-sEVs) on IVDD and explore the underlying molecular mechanisms	iMSC-sEV activates the Sirt6 pathway in vitro, resulting in rejuvenation of senescent NPCs and restoration of age-related functions. iMSC-sEV is highly enriched for miR-105-5p, which plays a key role in iMSC-sEV-mediated therapeutic effects by down-regulating the levels of the cAMP-specific hydrolase, PDE4D, and leading to Sirt6 activation	iMSC-sEVs could rejuvenate the senescence of NPCs and attenuate the development of IVDD
Dai et al. (2023)	An experimental study	Experimental in vivo and in vitro study	We investigate the use of PEVs as a therapeutic strategy for IVDD in this study	PEVs can restore impaired mitochondrial function, reduce oxidative stress, and restore cell metabolism by regulating the sirtuin 1 (SIRT1)-peroxisome proliferator-activated receptor gamma coactivator 1α (PGC1α)-mitochondrial transcription factor A (TFAM) pathway; in rat models, PEVs retard the progression of IVDD	The injection of PEVs can be a promising strategy for treating patients with IVDD
Diao et al. (2021)	An experimental study	Experimental in vitro study	Exploring the role of SIRT3 in regulating human stem cell homeostasis	SIRT3 expression is downregulated in senescent human mesenchymal stem cells (hMSCs). CRISPR/Cas9-mediated depletion of SIRT3 leads to impaired nuclear integrity, loss of heterochromatin, and accelerated senescence in hMSCs	SIRT3 has an important role in stabilizing heterochromatin and counteracting hMSC senescence
García-Sancho et al. (2017)	An experimental study	Clinical trial	Exploring the immune response to MSC in the treatment of osteoarthritis and lumbar spine disease	Immune response was weak and transient, with reactivity decaying during the first year. Consistently, better donor-recipient HLA matching did not enhance efficacy	This lack of reactivity is presumably due to the cooperation of 2 factors, (1) downregulation of the host immune responses by the transplanted MSCs and (2) effective insulation of these cells inside the articular cavity or the intervertebral disc, respectively
Pettine et al. (2017)	An experimental study	Clinical trial	The purpose of this study is to assess safety and feasibility of intradiscal bone marrow concentrate (BMC) injections to treat low back discogenic pain as an alternative to surgery with three year minimum follow-up	One-year MRIs showed improvement in a modified Pfirrmann classification in 40% of patients, and no patients had worsening imaging	There were no adverse events associated with bone marrow aspiration or injection, and this study provides evidence of the safety and feasibility of intradiscal BMC therapy
He et al. (2021b)	An experimental study	Experimental in vivo and in vitro study	To investigate the mechanisms by which hypoxia interacts with myeloid stem cell (NPSC) overload-induced cell death	Hypoxia exerted a protective effect on NPSCs under compression, partly by enhancing macroautophagy/autophagy.HIF1A overexpressing NPSCs showed greater resistance to overload-induced apoptosis in vitro	The anti-apoptotic effect of HIF1A on NPSCs under excessive mechanical loading suggests that restoration of hypoxia and manipulation of autophagy are essential for maintaining intrinsic repair and delaying disc degeneration

## Conclusion

Sirtuin family proteins can ameliorate the pathologic process of IVDD by regulating cellular senescence (Table 4), inflammation (Table 5), ECM (Table 6), apoptosis (Table 7), oxidative stress (Table 8), and mitochondrial function. Considering their important role in IVDD, these proteins can be explored as promising diagnostic biomarkers for IVDD. However, large-scale, multicenter prospective clinical trials are needed to validate the diagnostic value of sirtuins. These proteins are also involved in key aspects of senescence and apoptosis in myeloid cells. The continuous discovery of interacting molecules and the revelation of deep molecular mechanisms will facilitate the use of sirtuins in the prevention and treatment of IVDD in the future.

**Table 4:** Sirtuins and cellular senescence in IVDD

Authors (reference)	Type of study	Study design	Aim	Results	Conclusion
Stich et al. (2020)	An experimental study	Gene expression profiling and in vitro experiments	To assess whether cells from degraded AF are capable of initiating gene expression in extracellular matrix (ECM) molecular regeneration patterns	Mildly degraded natural AF tissue exhibited higher gene expression of common cartilage ECM genes. RTD-PCR analysis of BMP2 and TGFβ1-stimulated cells from mildly and severely degraded AF tissues showed increased expression of cartilage-related genes.TNFα stimulation increased the expression of MMP1, 3, and 13	Gene expression in natural AF tissues is different considering the grade of degeneration of the IVD
Li et al. (2019a)	An experimental study	In vitro study	This study aimed to investigate the specific role of Wnt/β-catenin signaling in compression-induced apoptosis, autophagy, and senescence in rat nucleus pulposus (NP) cells	compression elicited a time-dependent activation of Wnt/β-catenin signaling. The IWP-2 treatment decreased cell survival rate, which corresponded to downregulation of autophagy as well as increases in apoptosis and senescence. LiCl treatment enabled more efficient of cell survival accompanied by increased autophagy and downregulated apoptosis and senescence; however, in contrast to LiCl, overexpression of β-catenin aggravated compression-induced NP cells death	Moderate activation of Wnt/β-catenin signaling allows NP cells to survive more efficiently by down-regulating apoptosis, senescence, and up-regulating autophagy, whereas over-activation of Wnt/β-catenin signaling produces the opposite effect
Novais et al. (2019)	An experimental study	Experimental in vivo and in vitro study	Exploring the functional role of p16Ink4a in disc degeneration and aging	The cKO mice maintained expression of NP-cell phenotypic markers CA3, Krt19 and GLUT-1. Moreover, in cKO discs, levels of p19Arf and RB were higher without alterations in Ki67, γH2AX, CDK4 and Lipofuscin deposition. Interestingly, the cKO discs showed lower levels of SASP markers, IL-1β, IL-6, MCP1 and TGF-β1	p16Ink4a is dispensable for induction and maintenance of senescence, conditional loss of p16Ink4a reduces apoptosis, limits the SASP phenotype and alters matrix homeostasis of disc cells
Che et al. (2019)	An experimental study	A micro- and nano-level structural analysis of degenerative discs of rat tails	The aimed to assess the micro-nano structural characteristics of the degenerative disc to provide more specific biomechanical information than the Pfirrmann score	IVDD was observed microscopically at an earlier Pfirrmann grade (Pfirrmann II). As the Pfirrmann grade increased to III-V. In addition, the total GAG content of the nucleus pulposus decreased from an average of 640.33 μg GAG/ng DNA at the Pfirrmann grade I to 271.33 μg GAG/ng DNA at the Pfirrmann grade V. In the early stages of clinical degeneration of the discs (Pfirrmann grades II and III), compared with the inner layers, the mechanical properties of the outer annulus fibrosus underwent a significantly changed	The Pfirrmann grading system combined with intervertebral disc micro-nano structural changes more comprehensively reflected the extent of disc degeneration. These data may help improve our understanding of the pathogenesis and process of clinical disc degeneration
Wang et al. (2020a)	An experimental study	In vitro study using human IVD nucleus pulposus (NP) cells	The aimed to investigate the role of SIRT1 in IVDD by assessing the effects of SIRT1 overexpression on high-magnitude compression-induced senescence in NP cells	SIRT1-overexpression attenuated senescence and mitochondrial injury in NP cells subjected to high-magnitude compression. However, depletion of PINK1, a key mitophagic regulator, impaired mitophagy and blocked the protective role of SIRT1 against compression induced senescence in NP cells	SIRT1 plays a protective role in alleviating NP cell senescence and mitochondrial dysfunction under high-magnitude compression, the mechanism of which is associated with the regulation of PINK1-dependent mitophagy
Zhuo et al. (2021)	An experimental study	Experimental in vivo and in vitro study	The aim of this study was to investigate the biological role of SIRT1 in apoptosis and autophagy in rat NPCs under high-pressure stress and to determine whether the interaction between LC3B and Fas is involved in this process	High-magnitude compression aggravated cellular apoptosis and attenuated the expression levels of SIRT1 and microtubule-associated protein-1 light chain-3B (LC3B) in rat NPCs in a three-dimensional (3D) cell culture model and an in vivo rat tail compression model, whereas SIRT1 overexpression in NPCs partially reversed these indicators. Moreover, SIRT1 overexpression increased the formation of the LC3B/Fas complex, alleviated activation of the NF-κB pathway, and reduced NPC apoptosis. Finally, downregulation of LC3B partially activated the NF-κB pathway and aggravated NPC apoptosis	Upregulation of SIRT1 increased the formation of the LC3B/Fas complex, which inhibited NPC apoptosis by suppressing the NF-κB pathway under high compressive stress
Hao et al. (2022)	An experimental study	Experimental in vivo and in vitro study	The aim of this study was to investigate the potential role and pathophysiologic mechanisms of p300 in IDD	p300 increases FOXO3 expression by binding to the Sirt1 promoter, which contributes to the inactivation of the Wnt/β-catenin pathway. p300 slows the progression of IDD by disrupting the FOXO3/ Sirt1 axis of the Wnt/β-catenin pathway to slow down the progression of IDD. p300 is also known to be associated with the FOXO3/β-catenin pathway	p300 can inhibit IDD through a FOXO3-dependent mechanism
Wang et al. (2018a)	An experimental study	Experimental in vivo and in vitro study	The aim of this study was to explore the expression of SIRT3 in IVDD in vivo and in vitro and the role of SIRT3 in senescence, apoptosis and mitochondrial homeostasis under oxidative stress	The expression of SIRT3 decreased with IVDD, and SIRT3 knockdown reduced the tolerance of NPCs to oxidative stress. Honokiol (10 μM) improved the viability of NPCs under oxidative stress and promoted their properties of anti-oxidation, mitochondrial dynamics and mitophagy in a SIRT3-dependent manner. Furthermore, honokiol activated SIRT3 through the AMPK-PGC-1α signaling pathway	SIRT3 is involved in IVDD and showed the potential of the SIRT3 agonist honokiol for the treatment of IVDD
Lin et al. (2021a)	An experimental study	Experimental in vivo and in vitro study	The aim of this study was to investigate the effects and mechanisms of SIRT3 on NPC aging in vitro and in vivo	SIRT3 expression is reduced in degenerated NP tissues but increased in H2 O2 -induced NPC. Moreover, SIRT3 upregulation decreased oxidative stress, delayed senescence, and degeneration of NPC. In addition, activation of the AMPK/PGC-1α pathway can partially mitigate the NPC oxidative stress, senescence, and degeneration caused by SIRT3 knockdown. The study in vivo revealed that local SIRT3 overexpression can significantly reduce oxidative stress and ECM degradation of NPC, delay NPC senescence, thereby mitigating IVDD	SIRT3 mediated by the AMPK/PGC-1α pathway mitigates IVDD by delaying oxidative stress-induced NPC senescence

**Table 5:** Anti-inflammatory effects of sirtuins in IVDD

Authors (reference)	Type of study	Study design	Aim	Results	Conclusion
Jin et al. (2019)	An experimental study	in vitro study using human IVD nucleus pulposus (NP) cells	To investigate whether baicalein has a therapeutic effect on IDD by inhibiting the inflammatory response	Baicalein inhibited the overexpression of not only inflammatory cytokines, including NO, PGE2, TNF-α and IL-6, but also COX-2 and iNOS. Baicalein reversed IL-1β-induced overexpression of MMP13 and ADAMTS5 as well as degradation of aggregated glycans and type II collagen in a dose-dependent manner. Mechanistically, baicalein inhibited IL-1β-induced activation of the NF-κB and MAPK pathways. In addition, baicalein treatment improved puncture-induced IDD in a rat model	Baicalein has a therapeutic effect on IDD by inhibiting the inflammatory response
Han et al. (2019)	An experimental study	Rabbit annulus fibrosus stem cells (AFSCs) were treated with metformin and lipopolysaccharide (LPS) in vitro	The present study aimed to investigate the mechanism of intervertebral disc degeneration (IVDD) and identify an efficient treatment for low back pain	LPS induced HMGB1 release from the nuclei of AFSCs and caused cell senescence in a concentration-dependent manner. The production of PGE2 and HMGB1 was increased in the medium of the LPS-treated AFSCs. Certain inflammation-associated genes (IL-β1, IL-6, COX-2 and TNF-α) and proteins (IL-β1, COX-2 and TNF-α) and specific catabolic genes (MMP-3 and MMP-13) exhibited increased expression in LPS-treated AFSCs. However, the expression levels of other anabolic genes, such as collagen I and collagen II were decreased in LPS-treated AFSCs. Following addition of metformin to LPS-containing medium, HMGB1 was retained in the nuclei of AFSCs and the production of PGE2 and HMGB1 was reduced	The findings indicated that metformin exerted an anti-inflammatory effect by blocking the HMGB1 translocation and by inhibiting catabolic production and cell senescence in AFSCs
Cai et al. (2017)	An experimental study	In vitro study	To investigate the effect of a mixture of factors secreted by degenerating disc cells on transplanted exogenous healthy NPCs	The MAPK and NF-κB pathways were implicated in dCM-mediated responses of healthy NPCs. TGF-β1 partially reversed the dCM-mediated NPC dysfunction. Increased levels of inflammatory factors and decreased TGF-β1 levels in dCM suggest an inflammatory environment in degenerated disc tissue	The catabolic effect of dCM on human healthy NPCs is mediated by MAPK and NF-κB pathways and can be reduced by TGF-β1
Mouser et al. (2019)	Comparative Study	Experimental in vivo and in vitro study	The aim of this study was to investigate whether decreased tonicity under restricted swelling conditions (as occurring in early disc degeneration) could initiate an inflammatory cascade that mediates further degeneration	The extracellular environment directly affects NP cells instead of inducing a classical inflammatory cascade. Furthermore, IL-8 increased in swelling restricted samples, while IL-6 and PGE2 were elevated in free swelling controls	The involvement of different mechanisms in disc degeneration with intact AF compared to herniation
Miyamoto et al. (2000)	Comparative Study	Experimental in vivo and in vitro study	To elucidate the role of COX-2 in the pathogenesis of LDH radiculopathy	Lumbar disc herniated cells expressed mRNA for COX-2, IL-1β, and TNFα. disc-derived cells also produced large amounts of PGE2 by concomitant stimulation of inflammatory cytokines, and this PGE2 production was markedly inhibited by a selective inhibitor of COX-2,6-methoxy-2-naphthalenylacetic acid (6MNA)	COX-2 and inflammatory cytokines might play a causative role in the radiculopathy of LDH through upregulating PGE2 synthesis
Lowe et al. (1996)	An experimental study	In vitro study	To examined the effects of PGE1, PGE2, and PGE2 alpha on second messenger generation in relation to DNA and aggrecan synthesis in the nontransformed rat RCJ 3.1C5.18 (RCJ) chondrocyte cell line	The effects of PG on aggrecan production in RCJ cells appear to be regulated at the posttranscriptional level. Forskolin and (Bu)2cAMP mimicked the suppressive effects of PGE1 on [3H]TdR incorporation, as well as the stimulatory effect of PGE1 on aggrecan synthesis. In addition, the phorbol ester 12-O-tetradecanoyl phorbol acetate mimicked PGF2 alpha stimulation of [3H]TdR incorporation and aggrecan synthesis, and the effects of PGE2 alpha on these processes were blocked by protein kinase C inhibitors	In mammalian chondrocytes, PGE1 predominantly activates the cAMP protein kinase A second messenger system, PGE2 α predominantly affects the Ca2(+)-protein kinase C system, and PGE2 activates both pathways
Lin et al. (2018a)	An experimental study	Experimental in vivo and in vitro study	The severity of IVDD and the expression of inducible nitric oxide synthase (iNOS)/nitric oxide (NO) and cyclooxygenase (COX-2)/prostaglandin (PGE2) in Propionibacterium acnes-infected human intervertebral discs (IVDs) were quantified	The inhibition of iNOS/NO and COX-2/PGE2 activity ameliorated IVDD significantly, as evidenced by restored aggrecan and collagen II expression both in vivo and in vitro. Mechanistically, we found that *P. acnes* induced iNOS/NO and COX-2/PGE2 expressions via a reactive oxygen species- (ROS-) dependent NF-κB cascade	*P. acnes*-induced iNOS/NO and COX-2/PGE2 activation via the ROS-dependent NF-κB pathway is likely responsible for the pathology of IVDD
Chen et al. (2017)	An experimental study	Experimentalin vitro study	This study was conducted in order to investigate the function of IL-21 in intervertebral disc degeneration	The mRNA expression of ADAMTS-7, TNF-α, and MMP-13 was enhanced by IL-21 stimulation. mRNA expression of STAT-1, STAT-3, and STAT-5b was also enhanced by IL-21 treatment, with STAT-3 being the most significantly enhanced. The mRNA expression of TNF-α was significantly reduced after treatment with AG490 compared with treatment with IL-21 only	IL-21 is involved in the pathological development of IVD degeneration and IL-21 could aggravate IVD degeneration by stimulating TNF-α through the STAT signaling pathway
Gorth et al. (2019)	An experimental study	Experimental in vivo	To investigate the role of IL-1 in driving age-related disc degeneration	Knockout mice had significantly more degenerative changes in annular fibrosis (AF), as well as alterations in collagen type and maturation. at 20 months, there were no changes in nucleus pulposus (NP) extracellular matrix composition or cellular marker expression; however, IL-1KO NP cells accounted for a smaller proportion of NP compartments than in WT controls	Instead of protecting discs from age-related disc degeneration, global IL-1 deletion amplified the degenerative phenotype
Li et al. (2019b)	An experimental study	Experimentalin vitro study	The present study was aimed to study the effects of resveratrol on disc nucleus pulposus (NP) cell senescence in an inflammation environment	The inflammation group significantly increased SA-β-Gal activity and ROS content, decreased cell proliferation and telomerase activity, promoted G0/1 cell cycle arrest, up-regulated gene/protein expression of senescence markers (p16 and p53) and matrix catabolic metabolizing enzymes (MMP-3, MMP-13, and ADAMTS-4), and NP matrix macromolecules (aggregated glycans and collagen II) expression was down-regulated. However, resveratrol partially reversed the effects of inflammatory cytokines on these cellular senescence-related parameters	Resveratrol was effective to suppress cell senescence in an inflammatory environment
Guo et al. (2020)	An experimental study	Experimentalin vitro study	The aim of this study was to examine the role of circular RNA FAM169A (circ-FAM169A) in degenerative myeloid (NP) tissues and to verify its function in cultured human NP cells	Overexpression of circ-FAM169A in NP cells markedly enhanced extracellular matrix (ECM) catabolism and suppressed ECM anabolism in NP cells. Furthermore, circ-FAM169A sequestered miR-583, which could potentially upregulate BTRC, an inducer of the NF-κB signaling pathway	Circ-FAM169A promotes IDD development via miR-583/BTRC signaling
Yeung et al. (2004)	An experimental study	Experimentalin vitro study	The purpose of this study is that SIRT1 is a nicotinamide adenosine dinucleotide-dependent histone deacetylase that regulates the transcriptional activity of NF-kappaB	SIRT1 physically interacts with the RelA/p65 subunit of NF-kappaB and inhibits transcription by deacetylating RelA/p65 at lysine 310	SIRT1 activity augments apoptosis in response to TNFalpha by the ability of the deacetylase to inhibit the transactivation potential of the RelA/p65 protein
Lee et al. (2011)	An experimental study	Experimentalin vitro study	This study examined the role of SIRT1 in mediating heat stress and lipopolysaccharide (LPS)-induced immune and defense gene expression in HDPCs	LPS and heat stress synergistically increased the expression of SIRT1 and immune and defense genes. Resveratrol enhanced LPS- and heat stress-induced HO-1 and hBD-2 expression but decreased IL-8 messenger RNA levels. LPS stimulated HO-1 and hBD-2 messenger RNA expression, and heat stress inhibited sirtinol; SIRT1 small interfering RNA; and inhibitors of p38, ERK, JNK, and nuclear factor κB	SIRT1 mediates the induction of immune and defense gene expression in HDPCs by LPS and heat stress. SIRT1 may play a pivotal role in host immune defense system in HDPCS
Yoshizaki et al. (2010)	An experimental study	Experimental in vivo and in vitro study	Explore the role of SIRT1 in regulating pro-inflammatory pathways within macrophages	SIRT1 knockdown leads to increased expression of inflammatory genes. Treatment of Zucker adipose rats with SIRT1 activators greatly improves glucose tolerance, reduces hyperinsulinemia, and enhances systemic insulin sensitivity	SIRT1 as an important regulator of macrophage inflammatory responses in the context of insulin resistance
Yoshizaki et al. (2009)	An experimental study	Experimentalin vitro study	Exploring the potential effects of SIRT1 on insulin signaling pathways	SIRT1 expression was negatively correlated with inflammatory gene expression. In conclusion, we found that treatment of 3T3-L1 adipocytes with SIRT1 activators attenuated tumor necrosis factor α-induced insulin resistance	SIRT1 is a positive regulator of insulin signaling through, at least in part, its anti-inflammatory effects in 3T3-L1 adipocytes
Schug et al. (2010)	An experimental study	Experimental in vivo and in vitro study	Explore the important role played by SIRT1 in the immune response	Ablation of SIRT1 in macrophages hyperacetylates NF-κB, leading to increased transcriptional activation of pro-inflammatory target genes. Consistent with the increased expression of pro-inflammatory genes, Mac-SIRT1 KO mice attacked with a high-fat diet showed high levels of activated macrophages in the liver and adipose tissue, predisposing the animals to systemic insulin resistance and metabolic disorders	SIRT1 in macrophages functions to inhibit NF-κB-mediated transcription
Gillum et al. (2011)	An experimental study and clinical trial	Experimental in vivo study	To explore whether a reduction in SirT1 links overnutrition and adipose tissue inflammation	In vivo induced or genetic reduction of SirT1 leads to macrophage recruitment to adipose tissue, whereas overexpression of SirT1 prevents adipose tissue macrophage accumulation induced by prolonged high-fat feeding.SirT1 expression in human subcutaneous fat negatively correlates with adipose tissue macrophage infiltration	SirT1 regulates adipose tissue inflammation by controlling the gain of proinflammatory transcription in response to inducers such as fatty acids, hypoxia, and endoplasmic reticulum stress
Rajendrasozhan et al. (2008)	An experimental study and clinical trial	Experimentalin vitro study	To determine the expression of SIRT1 in lungs of smokers and patients with COPD, and to elucidate the regulation of SIRT1 in response to cigarette smoke in macrophages, and its impact on nuclear factor (NF)-kappaB regulation	Treatment of MonoMac6 cells with CSE showed decreased levels of SIRT1 associated with increased acetylation of RelA/p65 NF-kappaB. Mutation or knockdown of SIRT1 resulted in increased acetylation of nuclear RelA/p65 and IL-8 release, whereas overexpression of SIRT1 decreased IL-8 release in response to CSE treatment in MonoMac6 cells	SIRT1 plays a pivotal role in regulation of NF-kappaB-dependent proinflammatory mediators in lungs of smokers and patients with COPD
Lim et al. (2013)	An experimental study	Experimental in vivo and in vitro study	The purpose of this study was to determine (1) the effect of human preterm labor on SIRT6 expression in human gestational tissues and (2) the effect of SIRT6 inhibition using small interfering RNA (siRNA) on the pro-labor media	IL1B induced NFKB transcriptional activity. However, NFKB transcriptional activity was reduced when cells were also cotransfected with a vector expressing SIRT6	SIRT6 plays a role in regulating the terminal effector pathways of human labor and delivery via the NFKB pathway
Wu et al. (2019)	An experimental study	Experimental in vivo and in vitro study	This study focused on investigating SIRT6 expression in ACC and how it generates cancer phenotypes	Knockdown of SIRT6 promoted cell invasion, proliferation and migration, and inhibited cell death. In addition, SIRT6 knockdown was found to upregulate TLR4 and enhance phosphorylation of nuclear transcription factor-kappa B (NF-κB) subunit p65 as well as nuclear factor kappa-B kinase inhibitor. In addition, knockdown of SIRT6 significantly enhanced the expression of calcitonin gene-related peptide and transient receptor potential vanilloid isoform 1	SIRT6 serves as a tumor suppressor via regulation of the NF-κB pathway, which could offer an innovative strategy to treat ACC
Liu et al. (2017)	An experimental study	Experimental in vivo and in vitro study	To investigate whether Sirt6 prevents podocyte injury by epigenetically regulating Notch signaling	Sirt6 has pleiotropic protective effects in podocytes, including anti-inflammatory and anti-apoptotic effects, involvement in actin cytoskeleton maintenance and promotion of autophagy.Sirt6 also reduces expression of the urokinase fibrinogen activator receptor, a key factor in podocyte podocyte protrusion loss and proteinuria	Sirt6 represses Notch1 and Notch4 transcription by deacetylating histone H3K9
Ashburner et al. (2001)	An experimental study	Experimentalin vitro study	To investigate whether the trans-activation function of NF-kappaB is regulated through the interaction of the p65 (RelA) subunit with histone deacetylase (HDAC) co-repressor proteins	Inhibition of HDAC activity by tricostatin A (TSA) leads to an increase in basal and inducible expression of integrated NF-κB-dependent reporter genes. HDAC1 and HDAC2 target NF-kappaB through direct binding of HDAC1 to the Rel homology domain of p65. HDAC2 does not interact directly with NF-κB but can regulate NF-κB activity through binding to HDAC1. HDAC2 does not directly interact with NF-κB, but can regulate NF-κB activity by binding to HDAC1	The association of NF-kappaB with the HDAC1 and HDAC2 corepressor proteins functions to repress expression of NF-kappaB-regulated genes as well as to control the induced level of expression of these genes
Ma et al. (2014)	An experimental study	Experimentalin vitro study	To investigate the effect of jolkinolide B (JB), isolated from the roots of Morinda citrifolia, on NF-κB ligand receptor activator (RANKL)-induced osteoclast formation	JB inhibited RANKL-induced osteoclast differentiation from bone marrow macrophages (BMMs) without cytotoxicity. Furthermore, the expression of osteoclastic marker genes, such as tartrate-resistant acid phosphatase (TRAP), cathepsin K (CtsK), and calcitonin receptor (CTR), was significantly inhibited. JB inhibited RANKL-induced activation of NF-κB by suppressing RANKL-mediated IκBα degradation. Moreover, JB inhibited RANKL-induced phosphorylation of mitogen-activated protein kinases (p38, JNK, and ERK)	JB is an inhibitor of osteoclast formation
Li et al. (2014a)	An experimental study	Experimentalin vitro study	This study characterizes the potential to inhibit inflammatory cytokine production in degenerative disc (NP) cells by IL-10 and TGF-β treatment in a canine model of IDD	IL-10 and TGF-β treatment inhibited the expression of IL-1β and TNF-α and suppressed the development of inflammatory responses	IL-10 and TGF-β should be evaluated as therapeutic approaches for the treatment of lower back pain mediated by IDD
Wuertz et al. (1976)	An experimental study	Experimental in vivo and in vitro study	To determine whether resveratrol may be useful in treating nucleus pulposus (NP)-mediated pain	In vitro, resveratrol exhibited an anti-inflammatory and anticatabolic effect on the messenger RNA and protein level for IL-6, IL-8, MMP1, MMP3 and MMP13. This effect does not seem to be mediated via the MAP kinase pathways (p38, ERK, JNK) or via the NF-κB/SIRT1 pathway, although toll-like receptor 2 was regulated to a minor extent. In vivo, resveratrol significantly reduced pain behavior triggered by application of NP tissue on the dorsal root ganglion for up to 14 days	Resveratrol was able to reduce pro-inflammatory cytokine levels in vitro and showed analgesic potential in vivo
Song et al. (2020a)	An experimental study	Experimentalin vitro study	The aim of this study is to investigate the effect of DHP on nucleus pulposus (NP) cells in vitro	DHP inhibited IL-1β-induced up-regulation of ROS, TNF-α, IL-6, MMP-3, and ADAMTS-5. dHP significantly increased sirt1 and antioxidant protein SOD-1 levels. Furthermore, DHP significantly protected against IL-1β-induced degradation of collagen-II and aggregated glycans	DHP inhibited the ROS, inflammatory response and ECM degradation through activating Sirt1 in human NP cells
Cai et al. (2020)	An experimental study	Experimental in vivo and in vitro study	To explore whether Sirt1 inhibits MCP-1 in the intervertebral disc	MCP-1 was upregulated in the degenerated condition, which was opposite to Sirt1 expression. Res suppressed AP-1, the phosphorylation of c-Fos/c-Jun, and the MCP-1 expression. On the contrary, Sirt1 downregulation by Nico aggravated the phosphorylation of c-Fos/c-Jun and MCP-1 expression. However, the MCP-1 suppression did not affect the Sirt1 and AP-1 levels. The destruction of AP-1 activation also inhibited MCP-1 expression but not Sirt1. The upregulation of Sirt1 and suppression of MCP-1 improved the type II collagen expression and cell viability, which was injured by IL-1β	Sirt1 suppresses the MCP-1 production in the degenerated NP cells by suppressing the phosphorylation of the AP-1 subunits c-Fos and c-Jun
Li et al. (2022a)	An experimental study	Experimentalin vitro study	To investigate the effect of PPARβ/δ on COX-2 expression in the kidney	PPARβ/δ was functionally expressed in human mesangial cells (hMCs), where its expression was increased by interleukin-1β (IL-1β) treatment concomitant with enhanced COX-2 expression and prostaglandin E2 (PGE2) biosynthesis. PPARβ/δ could further augment the IL-1β-induced COX-2 expression and PGE2 production in hMCs. Moreover, both PPARβ/δ activation and overexpression markedly increased sirtuin 1 (SIRT1) expression	PPARβ/δ could augment the IL-1β-induced COX-2 expression and PGE2 production in hMCs via the SIRT1 pathway
Lim et al. (2015)	An experimental study	Experimentalin vitro study	The mechanism underlying Korean red ginseng water extract (KRG-WE) inhibition of hypoxia-induced COX-2 in human distal lung epithelial A549 cells	Hypoxia-induced COX-2 protein and mRNA levels and promoter activity were inhibited by KRG-WE. Hypoxia-induced cell migration was significantly reduced by KRG-WE. Inhibition of Sirt1 eliminated the effects of KRG-WE on hypoxia-induced COX-2 inhibition and cell invasion, suggesting that the inhibition was mediated by Sirt1	KRG-WE inhibits the hypoxic induction of COX-2 expression and cell invasion through Sirt1 activation
Kumar et al. (2021)	An experimental study	Experimental in vivo study	This study investigated the protective effects of melatonin on LPS-induced testicular nitro-oxidative stress, inflammation, and associated damages in the testes of male golden hamsters, Mesocricetus auratus	LPS upregulates NF-kB, COX-2, and iNOS expression to increase testicular inflammatory load, leading to decreased germ cell proliferation and survival, which ultimately leads to germ cell apoptosis, as shown by AO-EB staining and caspase-3 expression. Melatonin treatment upregulates testicular SIRT-1 expression to inhibit LPS-induced inflammatory proteins, i.e. NF-kB/COX-2/iNOS expression	Melatonin rescued testes from LPS-induced testicular nitro-oxidative stress, inflammation, and associated damages by upregulation of SIRT-1
Zhang et al. (2010)	An experimental study	Experimentalin vitro study	Whether SIRT1 deacetylates activator protein-1 (AP-1) to affect its transcriptional activity and target gene expression	SIRT1 directly interacts with the basic leucine zipper structural domains of c-Fos and c-Jun, the major components of AP-1, through which SIRT1 represses the transcriptional activity of AP-1. This process requires the deacetylase activity of SIRT1	SIRT1 may be a mediator of CR-induced macrophage regulation, and its deacetylase activity contributes to the inhibition of AP-1 transcriptional activity and COX-2 expression leading to amelioration of macrophage function
Kang et al. (2017)	An experimental study	Experimentalin vitro study	This study aimed to determine whether sirtuin 6 (SIRT6), a member of the sirtuin family of nicotinamide adenine dinucleotide-dependent deacetylases, protects the NP from ECM degradation in IDD	During the progression of IDD, SIRT6 expression was significantly reduced. overexpression of SIRT6 prevented IL-1β-induced degradation of the NP ECM, and RNA interference in NP cells resulted in SIRT6 depletion leading to ECM degradation. In addition, SIRT6 physically interacted with nuclear factor-κB (NF-κB) catalytic subunit p65, whose transcriptional activity was significantly inhibited by SIRT6 overexpression	SIRT6 prevented NP ECM degradation in vitro via inhibiting NF-κB-dependent transcriptional activity and that this effect depended on its deacetylase activity
Xie et al. (2022a)	An experimental study	Experimentalin vitro study	The purpose of the present study was to explore the effects of luteolin on Tumor necrosis factor (TNF)-α-induced inflammatory injury and senescence of human nucleus pulposus cells (HNPCs), as well as the underlying mechanisms of action of this compound	TNF-α induced a significant decrease in HNPC viability and an increase in the levels of inflammatory factors, whereas the application of lignans effectively increased cell viability and decreased intracellular interleukin (IL)-1β and IL-6 expression levels. In addition, lignocaine reduced apoptosis in a dose-dependent manner compared with the TNF-α group	Lignans inhibit TNF-α-induced inflammatory injury and senescence in HNPCs via the Sirt6/NF-κB pathway

**Table 6:** Sirtuins and extracellular matrix regulation

Authors (reference)	Type of study	Study design	Aim	Results	Conclusion
Emanuel et al. (2018)	An experimental study	Experimental in vivo and in vitro study	The aim to identify markers for disc degeneration and apply these to investigate early degenerative changes due to overloading and katabolic cell activity	In vivo, FTIR was more sensitive than biochemical and histologic analyses in identifying decreased proteoglycan content and increased collagen content in degenerative discs.FTIR analyses also revealed disturbances in the ECM as evidenced by increased collagen entropy. In isolation, the proteoglycan/collagen ratio was decreased by overloading and collagen entropy was increased	Matrix remodeling is the first detectable step towards intervertebral disc degeneration
Lu et al. (2021)	An experimental study	Experimental in vivo and in vitro study	To investigate the role of FGF21 in the progression of OA	FGF21 administration alleviated apoptosis, senescence, and extracellular matrix (ECM) catabolism of the chondrocytes induced by tert-butyl hydroperoxide (TBHP) by mediating autophagy flux. The FGF21-induced autophagy flux enhancement was mediated by the nuclear translocation of TFEB, which occurs due to the activation of the SIRT1-mTOR signaling pathway	FGF21 protects chondrocytes from apoptosis, senescence, and ECM catabolism via autophagy flux upregulation and also reduces OA development in vivo
Lin et al. (2021b)	An experimental study	Experimental in vivo and in vitro study	To explore the specific role of ECH in the occurrence and development of OA and its underlying mechanism in vivo and in vitro	ECH was shown to inhibit tert-butyl hydroperoxide (TBHP-)-induced OS and subsequently reduced the levels of p-PERK/PERK, GRP78, ATF4, p-eIF2α/eIF2α and CHOP in vitro. Meanwhile, ECH decreased MMP13 and ADAMTS5 levels and promoted Aggrecan and Collagen II levels, indicating ECM degradation inhibition. Furthermore, we found that ECH mediated its cellular effects by upregulating Sirt1	ECH can inhibit ER stress and ECM degradation by upregulating Sirt1 in mouse chondrocytes treated with TBHP
Liu et al. (2022)	An experimental study	Experimental in vivo and in vitro study	To explore whether the IL-4/JAK/STAT signaling pathway mediated macrophage polarization was involved in mechanical stimulation induced T-B healing	IL-4 production, activation of the JAK/STAT signaling pathway in macrophages, the ability of macrophages to polarize toward the M2 subtype, and T-B healing quality were significantly enhanced in the treadmill running group. This effect induced by mechanical stimulation was depleted after blockade of the IL-4/JAK/STAT signaling pathway	Mechanical stimulation could accelerate T-B healing via activating the IL-4/JAK/STAT signaling pathway that modulates macrophages to polarize towards M2 subtype
Zhao et al. (2022)	An experimental study	Experimental in vivo and in vitro study	To investigate the role of melatonin(MT) in OA rats	MT therapy protects articular cartilage in vivo	MT could reduce chondrocyte matrix degradation by up-regulating nuclear factor-kB (NF-κB) signaling pathway-dependent expression of SIRT1 and protecting chondrocyte by activating the TGF-β1/Smad2 pathway
Shang et al. (2022)	An experimental study	Experimental in vitro study	The study aims to investigate the possible role of paeonol in chondrocyte inflammation and cartilage protection in osteoarthritis (OA) as well as its regulation of SIRT1	Paeonol enhanced SIRT1 expression to inactivate the NF-κβ signaling pathway, thereby ameliorating inflammatory cytokine secretion, ECM degradation, and chondrocyte apoptosis	The present study confirm the potential of paeonol as a candidate OA drug
Eo et al. (2016)	An experimental study	Experimental in vitro study	To explore the mechanism of PEP-1-sirtuin (SIRT)2 induced dedifferentiation of articular chondrocytes	PEP-1-SIRT2 increased MMP-1 and -13 expression in a dose- and time-dependent manner, and PEP-1-SIRT2 increased phosphorylation of extracellularly regulated kinase (ERK); however, treatment of PD98059 with a mitogen-activated protein kinase inhibitor inhibited PEP-1-SIRT2-induced MMP-1 and -13 expression and dedifferentiation while restoring type II collagen expression in passaged 2 cells with type II collagen expression	PEP-1-SIRT2 promotes MMP-induced dedifferentiation via ERK signaling in articular chondrocytes
Smith et al. (2022)	An experimental study	Experimental in vitro study	To investigate the role of SIRT1 in chondrocyte development	Activation of SIRT1 resulted in a significant increase in ECM gene expression for collagen type II (COL2A1) and aggregated glycans (ACAN), as well as the chondrogenic transcription factors SOX5 and ARID5B	SIRT1 activation positively impacts on the expression of the main ECM proteins, while altering ECM composition and suppressing GAG content during human cartilage development
Kuai et al. (2022)	An experimental study	Experimental in vitro study	The purpose of the present study was to investigate lipopolysaccharide (LPS)-induced IDD progression in human nucleus pulposus cells (NPCs) and its potential mechanism	Evodiamine effectively alleviated LPS-induced NPCs apoptosis and caspase-3 activation and Evo treatment reversed the upregulation of matrix metalloproteinase-13, as well as the downregulation of collagen type II (collagen II), Sry-type high-mobility-group box 9 and aggrecan and reduced the production of pro-inflammatory factors TNF-α and IL-6 in LPS-stimulated NPCs	Evodiamine upregulated SIRT1 and inhibited LPS-induced NPCs apoptosis, extracellular matrix degradation and inflammation by activating the PI3K/Akt pathway
Ma et al. (2021)	An experimental study	Experimental in vitro study	To provide evidence that deacetylated-FOXO4 stabilizes chondrocyte (CH) extracellular matrix (ECM) related to SOX9 activation	FOXO4 protein transcriptionally activates SOX9 expression by binding to its promoter. Under the IL-1β stimulation, FOXO4 acetyl-lysine rate increased, and the SOX9 protein expression decreased, which was alleviated after the supplement of exogenic Sirt1 protein. Meanwhile, Sirt1 overexpression increased the collagen II and aggrecan and reduced the collagen I, collagen X, MMP-13, and ADAMTS-5 mRNA expression. However, the silencing of FOXO4 abolished the Sirt1 induced SOX9 expression and weakened the ECM production stability	FOXO4 acetylation aggravates during the degeneration of CHs, and the deacetylation of FOXO4 by Sirt1 could activate the SOX9 expression and result in maintaining the ECM stability of cartilage
Qian et al. (2022)	An experimental study	Experimental in vitro study	Tto investigate the role and mechanism of circ_0022383 on OA progression	Circ_0022383 was expressed at low levels in cartilage and IL-1β-induced primary chondrocytes from OA patients. circ_0022383 acted as a sponge for miR-3619-5p, which has been shown to target SIRT1. miR-3619-5p inhibition eliminated IL-1β-induced chondrocyte apoptosis, inflammation and ECM degeneration, which were counteracted by SIRT1 silencing	Circ_0022383 protected chondrocytes from IL-1β-induced apoptosis, inflammation and ECM degeneration by miR-3619-5p/SIRT1 axis, inspiring future therapy development for OA prevention
Wang et al. (2016b)	An experimental study	Experimental in vitro study	Investigating the role of SIRT1 in intervertebral disc inflammation	SIRT1 inhibited the induction of mRNA expression of proteases that degrade TNF-α-induced ECM. sIRT1 mRNA and protein expression was refractory to hypoxia and HIF-1α	SIRT1 is not affected by hypoxia and inflammatory cytokines in rat intervertebral discs
Qi et al. (2020)	An experimental study	Experimental in vitro study	To explored the effects and mechanisms of tyrosol on IDD progression in interleukin (IL)-1β-stimulated human nucleus pulposus cells (HNPCs)	Tyrosol attenuated IL-1β-induced reduction in viability, apoptosis and caspase-3/7 activity in HNPCs. Tyrosol treatment abrogated the increase in TNF-α, IL-6, NO, and PGE2 production in IL-1β-treated HNPCs.Sirt1 was upregulated by tyrosol, and Sirt1 silencing inhibited Akt phosphorylation in HNPCs. Sirt1 knockdown attenuated the effects of tyrosol on IL-1β-induced apoptosis, inflammation and ECM remodeling in HNPC	Upregulation of Sirt1 by tyrosol suppressed apoptosis and inflammation and regulated ECM remodeling in IL-1β-stimulated HNPCs through activation of PI3K/Akt pathway
Shen et al. (2016)	An experimental study	Experimental in vitro study	To investigate the effects of SIRT1 on proinflammatory stress and signal transduction pathways induced by interleukin-1β (IL-1β) in human degenerative nucleus pulposus (NP) cells	Direct regulation of SIRT1 expression did not affect the synthesis of extracellular matrix (ECM).SIRT1 overexpression mediated by the lentiviral vector suppressed IL-1β-induced ECM degradation and cell apoptosis	SIRT1 exerts anti-inflammatory effects on IL-1β-mediated NP cell degeneration through the TLR2/SIRT1/NF-κB pathway
Zhang et al. (2022)	An experimental study	Experimental in vivo and in vitro study	This study we aim to explore the function of Ori in IVDD pathological model	Ori treatment in vitro increased SIRT1/AMPK in NPCs, maintained ECM and ER balance and decreased oxidative stress (OS) response	Ori exerts its effects by upregulating AMPK and SIRT1
Xie et al. (2022b)	An experimental study	Experimental in vitro study	The purpose of the present study was to investigate the effect of hyperoside on tumor necrosis factor (TNF)-α-induced IDD progression in human nucleus pulposus cells (NPCs) and its potential mechanism	Hyperoside upregulated sirtuin-1 (SIRT1) and nuclear factor E2-related factor 2 (Nrf2) protein expression, and inhibition of SIRT1 or Nrf2 signaling reversed the protective effect of hyperoside on TNF-α-induced NPCs	Hyperoside ameliorated TNF-α-induced inflammation, extracellular matrix degradation, and endoplasmic reticulum stress-mediated apoptosis, which may be associated with the regulation of the SIRT1/NF-κB and Nrf2/antioxidant responsive element signaling pathways by hyperoside

**Table 7:** Regulation of apoptosis by sirtuins in IVDD

Authors (reference)	Type of study	Study design	Aim	Results	Conclusion
Liu et al. (2018a)	An experimental study	Experimental in vitro study	To investigate how SIRT1 attenuates oxidative stress-induced apoptosis	SIRT1 overexpression decreased the rate of apoptosis in human adipose stem cells (ADSCs), whereas SIRT1 down-regulation and EX527 showed the opposite effect.SIRT1 overexpression decreased total p53 protein, whereas SIRT1 down-regulation and EX527 increased the amount of p53 protein	SIRT1 had a pivotally protective role in the regulation of ADSCs aging and apoptosis induced by H2O2
Zhang et al. (2017)	An experimental study	Experimental in vitro study	To explore the mechanism by which oxidative stress promotes FoxO1 activity	Sirtuin 1 (SIRT1), a deacetylase that suppresses FoxO1 acetylation in GCs, was downregulated by miR-181a and reversed the promoting effects of H2O2 and miR-181a on FoxO1 acetylation and GC apoptosis	miR-181a mediates oxidative stress-induced FoxO1 acetylation and GC apoptosis by targeting SIRT1 both in vitro and in vivo
Brunet et al. (2004)	An experimental study	Experimental in vitro study	Probing the molecular mechanism of Sir2 life extension	SIRT1 had a dual effect on FOXO3 function: SIRT1 increased FOXO3's ability to induce cell cycle arrest and resistance to oxidative stress but inhibited FOXO3's ability to induce cell death	One way in which members of the Sir2 family of proteins may increase organismal longevity is by tipping FOXO-dependent responses away from apoptosis and toward stress resistance
Khan et al. (2012)	An experimental study	Experimental in vitro study	To investigate whether SIRT1 activators reduce oxidative stress and promote mitochondrial function in neuronal cells	The SIRT1 activators resveratrol (RSV) and SRTAW04 decreased ROS levels and promoted cell survival in RGC-5 cells and primary RGC cultures.The SIRT1 activators also increased succinate dehydrogenase (SDH) expression and promoted deacetylation of PGC-1α	SIRT1 activators prevent cell loss by reducing oxidative stress and promoting mitochondrial function in a neuronal cell line
Zhou et al. (2017)	An experimental study	Experimental in vivo study	To investigate the effects of SIRT1 on ICH injury and the underlying mechanisms	Activation of SIRT1 with SRT1720 (5 mg / kg) restored nuclear SIRT1, deacetylation of PGC-1α, and mitochondrial biogenesis and reduced mortality, behavioral deficits, and brain water content without significant changes in ICH-induced phosphorylated AMP-activated protein kinase (pAMPK). Activation of SIRT1 with SRT1720 also restored mitochondrial electron transport chain proteins and reduced apoptotic proteins in ICH	Activation of SIRT1 with SRT1720 (5 mg/kg) restored nuclear SIRT1, deacetylation of PGC-1α, and mitochondrial biogenesis and decreased mortality, behavioral deficits, and brain water content without significant changes in phosphorylated AMP-activated protein kinase (pAMPK) induced by ICH
Iaconelli et al. (2017)	An experimental study	Experimental in vitro study	Explore the extent to which reversible acetylation regulates AKT function	AKT is acetylated at Lys163 and Lys377 located in the kinase domain, two novel sites distinct from the acetylation sites in the PH-domain modulated by the sirtuins. Measurement of the functional effect of HDAC6 inhibition on AKT revealed decreased binding to PIP3, a correlated decrease in AKT kinase activity, decreased phosphorylation of Ser552 on β-catenin, and modulation of neuronal differentiation trajectories	Deacetylase activity of HDAC6 as a novel regulator of AKT signaling and point to novel mechanisms for regulating AKT activity with small-molecule inhibitors of HDAC6 currently under clinical development
Li et al. (2018a)	An experimental study	Experimental in vivo study	The current study was designed to investigate the role of PDE4 in EBI after SAH and explore the potential mechanism	PDE4 is predominantly located in neurons after SAH. rolipram attenuated brain edema and improved neurological function in the SAH rat model. In addition, rolipram increased the expression of Sirtuin1 (SIRT1) and upregulated Akt phosphorylation, which was accompanied by a decrease in neuronal apoptosis. Administration of sirtuinol inhibited Akt phosphorylation	PDE4 inhibition by rolipram protected rats against EBI after SAH via suppressing neuronal apoptosis through the SIRT1/Akt pathway
Zhu et al. (2021)	An experimental study	Experimental in vitro study	The aim of this study was to investigate whether m6A modification regulates TNF-α-mediated cell viability, cell cycle arrest, and cell senescence and how it works	miR-34a-5p was predicted to interact with the SIRT1 mRNA. SIRT1 overexpression counteracted the miR-34a-5p-promoted cell senescence. METTL14 participates in the TNF-α-induced m6A modification of miR-34a-5p to promote cell senescence in HNPCs and NP cells of IVDD patients. Downregulation of either METTL14 expression or miR-34a-5p leads to the inhibition of cell cycle arrest and senescence	SIRT1 mRNA is an effective binding target of miR-34a-5p, and SIRT1 overexpression mitigates the cell cycle arrest and senescence caused by miR-34a-5p
Xiang et al. (2020)	An experimental study	Experimental in vivo and in vitro study	The aimed to investigate the key role of circRNA in compression loading-induced IDD	CircRNA-CIDN was significantly downregulated in compression-treated human NP cells, and overexpressing circRNA-CIDN inhibited compression-induced apoptosis and NP ECM degradation. CircRNA-CIDN served as a sponge for miR-34a-5p, an important miRNA that enhanced compression-induced damage of NP cells via repressing the silent mating type information regulation 2 homolog 1 (SIRT1). CircRNA-CIDN was also verified to contain IDD development in an ex vivo IDD model	CircRNA-CIDN binding to miR-34a-5p played an important role in mitigating compression loading-induced nucleus pulposus cell damage via targeting SIRT1, providing a potential therapeutic strategy for IDD treatment
Xie et al. (2019)	An experimental study	Experimental in vivo and in vitro study	This study aimed to investigate the role of circular RNAs (circRNAs) in the pathogenesis of IVDD	Downregulation of circERCC2 increased the level of miR-182-5p and decreased the level of SIRT1 in degenerative NP tissues in vivo as well as in TBHP-stimulated NPCs in vitro. Treatment of SIRT1-si activated apoptosis and inhibited mitophagy. Moreover, miR-182-5p-si could regulate the mitophagy and the apoptosis of NPCs by targeting SIRT1. The effects of circERCC2 on NPCs and IVDD rat model were mediated by miR-182-5p/SIRT1 axis	First evidence that circERCC2 could ameliorate IVDD through miR-182-5p/SIRT1 axis by activating mitophagy and inhibiting apoptosis, and suggests that circERCC2 is a potentially effective therapeutic target for IVDD
Song et al. (2020b)	An experimental study	Experimental in vivo and in vitro study	Explore the mechanisms by which circular RNAs (circRNAs) regulate IDD	CircRNA_0000253 was selected as having the maximum upregulation in degenerative NPC exosomes. ceRNA analysis showed that circRNA_0000253 could adsorb miRNA-141-5p to downregulate SIRT1. circRNA_0000253 was confirmed to increase IDD by adsorbing miRNA-141-5p and downregulating SIRT1 in vivo and in vitro	Exosomal circRNA_0000253 owns the maximum upregulation in degenerative NPC exosomes and could promote IDD by adsorbing miRNA-141-5p and downregulating SIRT1
Chen et al. (2021a)	An experimental study	Experimental in vivo and in vitro study	Explore the molecular mechanisms of IDD	MiR-22-3p plays a mechanistic role in the development of IDD by targeting SIRT1, which in turn activates the JAK1/STAT3 signaling pathway. Therapeutically, the delivery of miR-22-3p inhibitors and mimics through the synthesized nanoparticles in the IDD model alleviates and aggravates IDD, respectively	The nanocarriers enhance transportation of miR-22-3p to nucleus pulposus cells, thus enabling the in vivo inhibition of miR-22-3p for therapeutic purposes and consequently promoting the development of miRNA-specific drugs for IDD
Wang et al. (2016c)	An experimental study	Experimental in vivo and in vitro study	Exploring miR-141 as an important molecular mechanism for IDD	MiR-141 is a key regulator of IDD. miR-141 drives IDD by inducing apoptosis in myeloid nucleus pulposus (NP) cells. In addition, miR-141 KO in mice attenuated spontaneous and surgically-induced IDD. Mechanistically, miR-141 promotes IDD by targeting and depleting the negative regulator of the NF-κB pathway, SIRT1. Therapeutically, up- or down-regulation of miR-141 by nanoparticle delivery in IDD models exacerbated or alleviated experimental IDD, respectively	MiR-141 promotes IDD progression in part by interacting with the SIRT1/NF-κB pathway
Chen et al. (2023a)	An experimental study	Experimental in vivo and in vitro study	To demonstrate that miR-22-3p is essential in the regulation of IDD	MiR-22-3p plays a mechanistic role in the development of IDD by targeting SIRT1, which in turn activates the JAK1/STAT3 signaling pathway. This is confirmed by a luciferase reporter assay and western blot analysis. Therapeutically, the delivery of miR-22-3p inhibitors and mimics through the synthesized nanoparticles in the IDD model alleviates and aggravates IDD, respectively	The nanocarriers enhanced the translocation of miR-22-3p to myeloid cells, which enabled the in vivo inhibition of miR-22-3p for therapeutic purposes, thus facilitating the development of miRNA-specific drugs
Ji et al. (2018)	An experimental study	Experimental in vivo and in vitro study	Understand the molecular mechanisms that regulate disc maintenance and destruction	miR-141 drives IDD by inducing apoptosis in nucleus pulposus (NP) cells. miR-141 promotes IDD by targeting and depleting SIRT1, a negative regulator of the NF-κB pathway. miR-141 also promotes IDD by targeting and depleting SIRT1, a negative regulator of the NF-κB pathway. Therapeutically, up- or down-regulation of miR-141 by nanoparticle delivery in IDD models exacerbated or alleviated experimental IDD, respectively	MiR-141 promotes IDD progression in part by interacting with the SIRT1/NF-κB pathway
Meng et al. (2023)	An experimental study	Experimental in vivo and in vitro study	The aim was to verify the potential therapeutic mechanisms of miR-106b-5p for IDD	Overexpression of miR-106b-5p in NP cells decreases cell growth, induces apoptosis, hinders extracellular matrix formation, and increases the expression of matrix-degrading enzymes through the SIRT2/MAPK/ERK signaling pathway	Targeting miR-106b-5p in intervertebral disc has therapeutic effects on IDD
Liao et al. (2019)	An experimental study	Experimental in vivo and in vitro study	To explore the mechanism by which MSC-exos inhibits excessive NP cell apoptosis during IDD	MSC-exos could attenuate ER stress-induced apoptosis by activating AKT and ERK signaling. Moreover, delivery of MSC-exos in vivo modulated ER stress-related apoptosis and retarded IDD progression in a rat tail model	MSC-exos can regulate ER stress-induced apoptosis during IVD degeneration associated with AGEs
Liu et al. (2020a)	An experimental study	Experimental in vitro study	To investigate the effects of diabetes-related hyperglycemia on NPMSC biology	High glucose concentration (HG-NPMSC) stemness gene expression as well as mRNA and protein expression of silencing information regulatory protein 1 (SIRT1), SIRT6, hypoxia-inducible factor-1α (HIF-1α), and glucose transporter protein 1 (GLUT-1) were significantly decreased, whereas apoptosis, cellular senescence, and cysteine asparagin-3 expression were increased	High glucose concentration significantly decreased cell proliferation, colony formation ability, migration and wound-healing capability of nucleus pulposus-derived mesenchymal stem cells

**Table 8:** Oxidative stress management by sirtuins

Authors (reference)	Type of study	Study design	Aim	Results	Conclusion
Li et al. (2022b)	An experimental study	Experimental in vivo and in vitro study	Explore the role of ATF3 in IDD	ATF3 positively regulates tert-butyl hydroperoxide (TBHP-)-induced nucleus pulposus cell (NPC) apoptosis, ROS production, inflammatory response, and extracellular matrix (ECM) degradation.ATF3 is a direct target of miR-874-3p, suggesting that up-regulation of ATF3 in IDD may be due, at least in part, to down-regulation of miR-874-3p in IDD, which alleviates the inhibition of ATF3 by miR-874-3p. -874-3p inhibition of ATF3	ATF3 has the potential to be used as a promising therapeutic target against IDD
Zheng et al. (2019)	An experimental study	Experimental in vivo and in vitro study	This study was performed to confirm whether TFEB was involved in IVDD development and its mechanism	The nuclear localization of TFEB declined in degenerated rat NP tissue as well as in TBHP treated NP cells.TFEB overexpression ameliorates the puncture-induced IVDD development in rats	TFEB overexpression suppressed TBHP-induced apoptosis and senescence via autophagic flux stimulation in NP cell and alleviates puncture-induced IVDD development in vivo
Chen et al. (2019)	An experimental study	Experimental in vivo and in vitro study	To study the effect of melatonin on IDD	Melatonin reduces apoptosis induced by tert-butyl hydroperoxide in nucleus pulposus (NP) cells. Melatonin was shown to preserve the extracellular matrix (ECM) content of collagen II, Aggrecan, and Sox-9, while inhibiting the expression of matrix denaturing enzymes, including MMP-13 and ADAMTS-5	Melatonin protected NP cells against apoptosis via mitophagy induction and ameliorated disc degeneration, providing the potential therapy for IDD
Li et al. (2018b)	An experimental study	Experimental in vitro study	To investigate the effect of SIRT1 on the invasiveness and inflammatory response of cultured RA-FLS	Overexpression of SIRT1 significantly inhibited RA-FLS proliferation, migration and invasion, and SIRT1 overexpression also significantly increased RA-FLS apoptosis and caspase-3 and -8 activity. Focusing on the inflammatory phenotype, we found that SIRT1 significantly reduced RA-FLS secretion of TNF-α, IL-6, IL-8 and IL-1β. Mechanistic studies further revealed that SIRT1 inhibits the NF-κB pathway by reducing p65 protein expression, phosphorylation and acetylation in RA-FLS	SIRT1 is a key regulator in RA pathogenesis by suppressing aggressive phenotypes and inflammatory response of FLS
Yao et al. (2018)	An experimental study	Experimental in vitro study	In this study, we investigated the role of SIRT1 in the FoxO1/β-catenin signaling pathway in oxidative stress and its mechanism in an osteoblast progenitor cell line (MC3T3-E1)	OB apoptosis and elevated oxidative stress in cells were simulated by H2O2, which was inhibited by moderate SIRT1 overexpression through reducing the oxidative stress. FOXO1 and β-catenin pathway activity was downregulated by SIRT1 and eventually resulted in inhibition of target genes, including the proapoptotic gene B cell lymphoma-2 interacting mediator of cell death, DNA repair gene growth arrest and DNA damage inducible protein 45 and the OB differentiation suppressor gene peroxisome proliferator activated receptor (PPAR)-γ. Furthermore, β-catenin and PPAR-γ were inhibited by SIRT1	Moderate overexpression of SIRT1 (~ threefold of normal level) may directly or indirectly inhibit apoptosis of OBs via the FOXO1 and β-catenin signaling pathway
Lin et al. (2018b)	An experimental study	Experimental in vitro study	The role of Sirt1/p53 in the protective effect of berberine (BBR) against hypoxia/reoxygenation (H/R)-mediated mitochondrial dysfunction in rat renal tubular epithelial cells (NRK-52E cells) was investigated	Pretreatment with BBR increased cell viability and inhibited mitochondrial oxidative stress and apoptosis. protein expression of Sirt1 was also enhanced with the reduction of p53. In addition, nuclear translocation of p53 and its acetylation were inhibited in NRK-52E cells pretreated with BBR. However, knockdown of Sirt1 counteracted the renoprotective effect of BBR	BBR preconditioning protects rat renal tubular epithelial cells against H/R-induced mitochondrial dysfunction via regulating the Sirt1/p53 pathway
Wang et al. (2019b)	An experimental study	Experimental in vivo study	The ability of the sirtuin-1 (SIRT1) agonist SRT1720 to reduce cognitive decline in type 2 diabetes mellitus (T2DM) was studied	SRT1720 significantly increased body weight, decreased FBG, improved cognitive function and reduced the levels of proteins associated with oxidative stress and inflammation damage in T2DM rats. Additionally, SRT1720 significantly decreased NF-κB p65 mRNA expression and increased eNOS and PPARγ expression. SRT1720 significantly reduced caspase-3 activity and HSP70 protein expression, and increased p-AMPK, SIRT1, Nrf2 and HO-1 protein expression	SRT1720 may reduce cognitive decline in T2DM rats through antioxidative and anti-inflammatory action via NF-κB and AMPK-dependent mechanisms
Shin et al. (2017)	An experimental study	Experimental in vivo study	To study the hepatoprotective mechanism of genipin in IR-induced liver injury, with a special focus on mitochondrial quality control (QC)	Hepatic IR decreased the levels of mitochondrial biogenesis related proteins (e.g., peroxisome proliferator-activated receptor gamma coactivator 1α, nuclear respiratory factor 1, and mitochondrial transcription factor A), mitophagy related proteins (e.g., Parkin), and fusion related protein (e.g., mitofusin 2). Furthermore, hepatic IR decreased the levels of sirtuin1 protein and phosphorylation of AMP-activated protein kinase	Genipin protects against IR-induced hepatic injury via regulating mitochondrial QC
Ding et al. (2015)	An experimental study	Experimental in vivo study	To investigate the effect of upregulation of SIRT1 in the diabetic heart on susceptibility to ischemic injury	Upregulation of SIRT1 in diabetic hearts improved cardiac function and reduced infarct size to the same extent as in nondiabetic animals after MI/R, which was associated with reduced serum creatine kinase-MB, lactate dehydrogenase activity, and cardiomyocyte apoptosis.Ad-SIRT1 increased eNOS phosphorylation and decreased eNOS acetylation in diabetic hearts.The NOS inhibitor L-NAME inhibited SIRT1-enhanced eNOS phosphorylation and attenuated SIRT1-mediated antiapoptotic and antioxidant effects, as well as cardioprotective effects. The NOS inhibitor L-NAME inhibited the enhanced eNOS phosphorylation by SIRT1 and attenuated the SIRT1-mediated antiapoptotic and antioxidant effects as well as cardioprotective effects	Overexpression of SIRT1 reduces diabetes-exacerbated MI/R injury and oxidative stress via activating eNOS in diabetic rats
Jeong et al. (2007)	An experimental study	Experimental in vitro study	Probing SIRT1 attenuates apoptotic responses through deacetylation	SIRT1 enhances DNA repair and deacetylation of the repair protein Ku70. Ectopic overexpression of SIRT1 leads to increased repair of radiation-generated DNA strand breaks. In addition, SIRT1 physically complexes with the repair protein Ku70, leading to subsequent deacetylation. Dominant-negative SIRT1 is a catalytically inactivated form that does not induce deacetylation of the Ku70 protein and an increase in DNA repair capacity	SIRT1 modulates DNA repair activity, which could be regulated by the acetylation status of repair protein Ku70 following DNA damage
Xiong et al. (2011)	An experimental study	Experimental in vivo study	Explore the functional consequences of the interaction between FoxO1 and SIRT1	oxO1 directly activates SIRT1 promoter activity, and both IRS-1 and FKHD-L enable FoxO1-dependent SIRT1 transcription. FoxO1 binds to the IRS-1 and FKHD-L sites of the SIRT1 promoter. Consistently, FoxO1 overexpression increases SIRT1 expression, whereas depletion of FoxO1 by siRNA decreases SIRT1 expression at messenger RNA and protein levels in vascular smooth muscle cells and HEK293 cells	Positive feedback mechanisms regulate FoxO1-dependent SIRT1 transcription and suggest a previously unappreciated function of FoxO1
Kim et al. (2015)	An experimental study	Experimental in vivo study	Mechanisms by which CO regulates hepatic I/R injury	CO increased SIRT1 expression by inhibiting miR-34a. both CO and PFT reduced proinflammatory cytokine production in vitro. Knockdown of SIRT1 in LPS-stimulated macrophages increased NF-κB acetylation and increased proinflammatory cytokines.CO treatment decreased miR-34a expression and increased SIRT1 expression in oxidant-attacked hepatocytes; and rescued SIRT1 expression in cells transfected with either p53 or miR-34a	In response to CO, enhanced SIRT1 expression mediated by miR-34a inhibition protects against liver damage through p65/p53 deacetylation
Iacovelli et al. (2016)	An experimental study	Experimental in vitro study	This study examines the ability of PGC-1α to regulate RPE metabolic program and oxidative stress response	Maturation of ARPE-19 and hfRPE was associated with significant increase in mitochondrial mass, expression of oxidative phosphorylation (OXPHOS) genes, and PGC-1α gene expression. Overexpression of PGC-1α increased expression of OXPHOS and fatty-acid β-oxidation genes, ultimately leading to the potent induction of mitochondrial respiration and fatty-acid oxidation	This study provides important insights into the metabolic changes associated with RPE functional maturation and identifies PGC-1α as a potent driver of RPE mitochondrial function and antioxidant capacity
Liang et al. (2020)	An experimental study	Experimental in vitro study	To investigate the functional role of the SIRT1/PGC-1α pathway in the regulation of autophagy/mitochondrial autophagy and tight junction protein expression in porcine intestinal epithelial cell (IPEC-1) oxidative dysfunction	H2O2 exposure resulted in high accumulation of ROS, decreased mitochondrial membrane potential and inhibition of tight junction molecules in IPEC-1 cells. In addition, COX IV mRNA expression and the SIRT1/PGC-1α pathway were also inhibited, and SIRT1 activation significantly suppressed ROS production, leading to increased mitochondrial membrane potential and COX IV expression	Autophagy/mitophagy elevation caused by SIRT1/PGC-1α pathway activation might be a protective mechanism to increase tight junction integrity against oxidative stress-mediated ROS production in IPEC-1 cells
Zhang et al. (2013)	An experimental study	Experimental in vivo study	The study investigated the effect of nuclear factor erythroid 2-related factor 2 (Nrf2), a cellular oxidative stress sensor, on energy homeostasis and liver pathophysiology during fasting	Fasting reduced liver size in Nrf2-expressing mice but not in Nrf2 null mice. Nrf2 null mice accumulated more nonesterified free fatty acids and triglycerides in the liver after fasting compared with mice of other genotypes. It is expected that increased oxidative stress in the liver of Nrf2-null mice would lead to mitochondrial damage, which would reduce the increased oxidation and lipid accumulation in the liver of Nrf2-null mice	The Nrf2-regulated signaling pathway is critical in protecting mitochondria from oxidative stress during feed deprivation, which ensures efficient utilization of fatty acids in livers of mice
Huang et al. (2015)	An experimental study	Experimental in vitro study	To investigate the effect of RAGE (specific receptor for AGEs) on Sirt1 in terms of protein expression and deacetylase activity	Along with reduced expression of RAGE, the specific receptor for AGEs, Polydatin (PD) significantly reversed the down-regulation of Sirt1 in protein expression and deacetylase activity and attenuated FN and TGF-β1 expression in GMCs exposed to AGEs	The resistance of PD on upregulated FN and TGF-β1 induced by AGEs via oxidative stress in GMCs is closely associated with its activation of Sirt1-Nrf2-ARE pathway
Liu et al. (2018b)	An experimental study	Experimental in vitro study	The present study aimed to explore the possible molecular mechanism underlying the anti-apoptosis and protective effects of resveratrol (RES) on the co-culture of Sertoli-germ cells and rat testes	Microcystin-leucine-arginine (MC-LR) treatment inhibited proliferation and induced apoptosis in Sertoli-germ cells. In addition, SIRT1 and Bcl-2 were inhibited, whereas p53 and Ku70 acetylation, Bax expression and cleaved caspase-3 were up-regulated by MC-LR. However, RES pretreatment ameliorated MC-LR-induced apoptosis and SIRT1 inhibition and down-regulated MC-LR-induced increases in p53 and Ku70 acetylation, Bax expression and caspase-3 activation	The administration of RES could ameliorate MC-LR-induced Sertoli-germ cell apoptosis and protect against reproductive toxicity in rats by stimulating the SIRT1/p53 pathway, suppressing p53 and Ku70 acetylation and enhancing the binding of Ku70 to Bax
Jacobs et al. (2008)	An experimental study	Experimental in vitro study	Whether SIRT3 interacts with and regulates the activity of FOXO proteins	Overexpression of the wild-type SIRT3 gene increases FOXO3a DNA binding activity as well as FOXO3a-dependent gene expression. Biochemical analysis of HCT116 cells overexpressing the deacetylated mutant compared to the wild-type SIRT3 gene indicated an overall oxidized intracellular environment monitored by increased intracellular superoxide and oxidized glutathione levels	SIRT3 and FOXO3a constitute a potential mitochondrial signaling cascade response pathway
Sundaresan et al. (2009)	An experimental study	Experimental in vivo and in vitro study	Explore how Sirt3 comes to protect the mouse heart	Sirt3 blocks cardiac hypertrophy by activating the forkhead box O3a-dependent (Foxo3a-dependent), antioxidant-encoding genes manganese superoxide dismutase (MnSOD) and catalase (Cat), thereby decreasing cellular levels of ROS. Reduced ROS levels inhibit Ras activation and downstream signaling through the MAPK/ERK and PI3K/Akt pathways. This leads to inhibition of the activity of transcription factors (especially GATA4 and NFAT) and translation factors (especially eukaryotic initiation factor 4E (elf4E) and S6 ribosomal protein (S6P), which are involved in the development of cardiac hypertrophy	SIRT3 is an endogenous negative regulator of cardiac hypertrophy
Tseng et al. (2013)	An experimental study	Experimental in vitro study	Elucidate the interaction between FOXO3 is SIRT3	Hydrogen peroxide induces SIRT3 to deacetylate FOXO3 at K271 and K290, which then upregulates a set of genes critical for mitochondrial homeostasis (mitochondrial biogenesis, fission/fusion, and mitochondrial autophagy)	SIRT3 deacetylates FOXO3 to protect mitochondria against oxidative stress provides a possible direction for aging-delaying therapies and disease intervention
Valle et al. (2005)	An experimental study	Experimental in vitro study	It is hypothesized that the transcriptional coactivator, peroxisome proliferator-activated receptor-gamma coactivator 1alpha (PGC-1alpha), a major regulator of oxidative metabolism and mitochondrial biogenesis, may be involved in the transcriptional regulation of mitochondrial antioxidant defense systems in vascular endothelial cells	Endothelial cells overexpressing PGC-1alpha showed reduced accumulation of reactive oxygen species (ROS), increased mitochondrial membrane potential, and reduced apoptotic cell death. siRNA down-regulation of PGC-1alpha levels decreased the expression of mitochondrial detoxification proteins	PGC-1α may play a crucial protective role in the vascular complications of diabetes, in which mitochondrial metabolism of glucose has been shown to lead to oxidative stress and vascular endothelial cell dysfunction
Pillai et al. (2015)	An experimental study	Experimental in vivo study	Exploring HKL blockade agonist-induced and pressure overload-mediated cardiac hypertrophic responses	The presence of HKL in mitochondria nearly triples Sirt3 expression and suggests that HKL may bind to Sirt3 to further increase its activity.Increased Sirt3 activity is associated with decreased acetylation of mitochondrial Sirt3 substrates, MnSOD, and Oligomycin Sensitivity Conferring Protein (OSCP)	HKL is a pharmacological activator of Sirt3 capable of blocking, and even reversing, the cardiac hypertrophic response
Kong et al. (2010)	An experimental study	Experimental in vitro study	To investigate the molecular mechanism by which PGC-1alpha induces several key reactive oxygen species (ROS) detoxification enzymes	Knockdown of PGC-1alpha results in reduced Sirt3 gene expression.PGC-1alpha co-localizes with ERRalpha in the mSirt3 promoter. Knockdown of ERRalpha reduced induction of Sirt3 by PGC-1alpha in C(2)C(12) myotubes.Overexpression of SIRT3 or PGC-1alpha in C(2)C(12) myotubes reduced basal ROS levels	Sirt3 acts as a downstream target gene of PGC-1α and mediates the effects of PGC-1α on cellular ROS production and mitochondrial biogenesis
Zhu et al. (2023)	An experimental study	Experimental in vivo and in vitro study	Exploring the effects of crosstalk between oxidative stress and iron death in IVDD	Sirt3 is reduced and iron death occurs after IVDD. Knockdown of Sirt3 (Sirt3) promotes IVDD and poor pain-related behavioral scores by increasing oxidative stress-induced iron death. overexpression of USP11 significantly ameliorates oxidative stress-induced iron death, thereby alleviating IVDD by increasing Sirt3	USP11-mediated oxidative stress-induced iron death has been identified as a promising target for the treatment of IVDD
Song et al. (2018)	An experimental study	Experimental in vivo and in vitro study	The study examined whether the accumulation of AGEs exacerbated NP cell apoptosis and IVD degeneration and explored the mechanisms behind these effects	AGEs treatment significantly inhibited human NP cell viability and proliferation, which was mainly due to apoptosis.Impairment of Sirtuin3 (SIRT3) function and mitochondrial antioxidant network is an important mechanism of AGEs-induced oxidative stress and secondary human NP cell apoptosis	SIRT3 prevents AGEs-induced apoptosis and IVD degeneration in human NP cells
Zhou et al. (2019)	An experimental study	Experimental in vivo and in vitro study	The objective of this paper is to determine whether SIRT3 could retard intervertebral disc degeneration and study the mechanism	SIRT3 expression was reduced in the degenerating human nucleus pulposus.Nucleus pulposus cells with SIRT3 overexpression vectors expressed more collagen II.FOXO3a and superoxide dismutase 2 (SOD2), suggesting that SIRT3 may ameliorate intervertebral disc degeneration through anti-oxidative stress	SIRT3 is a protective factor for intervertebral discs and can reduce oxidative stress in the intervertebral disc
Hu et al. (2021)	An experimental study	Experimental in vivo and in vitro study	In this study, we investigated the roles of the Nrf2/Sirt3 pathway and tert-butylhydroquinone (t-BHQ) in IVDD and elucidated their potential working mechanisms	Activation of the Nrf2/Sirt3 pathway inhibited tert-butyl hydroperoxide- (TBHP-) induced apoptosis and mitochondrial dysfunction in vitro. In addition to apoptosis, upregulation of the Nrf2/Sirt3 pathway induced by t-BHQ restored TBHP-induced autophagic flux disturbances. However, its protective effect was reversed by chloroquine and Si-ATG5	The Nrf2/Sirt3 pathway and its agonist represent a potential candidate for treating IVDD
Liu et al. (2023)	An experimental study	Experimental in vitro study	The mechanism by whichDuhuo Jisheng Decoction( DHJSD) prevents IVD degeneration in IL-1β-treated human myeloid cells in vitro was investigated	DHJSD enhanced the viability of NP cells treated with IL-1β in a concentration–time-dependent manner.DHJSD treatment could effectively delay IL-1β-induced NP apoptosis by affecting the miR-494/SIRT3/mitochondrial autophagy signaling axis	MiR-494/SIRT3/mitophagy signaling pathway is responsible for the apoptosis and mitochondrial dysfunction of NP cells and that DHJSD may exert protective effects against IVD degeneration by regulating the miR-494/SIRT3/mitophagy signal axis
Wang et al. (2019c)	An experimental study	Experimental in vitro study	Investigating the inhibitory properties of metformin on mitochondrial damage	Metformin treatment upregulated SIRT3 expression and attenuated the loss of cell viability and reduced mitochondria-induced ROS production in IL-1β-stimulated chondrocytes. Metformin also attenuated the expression of IL-1β-induced catabolic genes, and the IRT3 inhibitor 3-TYP effectively inhibited the initiation of mitochondrial autophagy as a result of reduced expression of PINK1 and Parkin, decreased LC3II/LC3I, enhanced expression of MMP3 and MMP13, and decreased expression of collagen II	Metformin inhibits IL-1β-induced oxidative and osteoarthritis-like inflammatory changes through enhancement of the SIRT3/PINK1/Parkin signaling pathway

Although SIRTs have potential advantages in preventing and treating IVDD through the regulation of multiple pathways, they also have the following limitations: first, most of the experiments on the regulation of multiple pathways by SIRTs for the treatment of IVDD are mainly in vitro experiments, so there is a lack of in-depth understanding of the mechanism of in vivo action of SIRTs for the treatment of IVDD; second, the research on the treatment of IVDD by SIRTs-related activators is still in the experimental stage. Therefore, more high-quality animal and preclinical studies are needed to verify their efficacy and safety.

## Data Availability

Not applicable.
